# Beta-blockers disrupt mitochondrial bioenergetics and increase radiotherapy efficacy independently of beta-adrenergic receptors in medulloblastoma

**DOI:** 10.1016/j.ebiom.2022.104149

**Published:** 2022-07-08

**Authors:** Maïlys Rossi, Julie Talbot, Patricia Piris, Marion Le Grand, Marie-Pierre Montero, Mélanie Matteudi, Emilie Agavnian-Couquiaud, Romain Appay, Céline Keime, Daniel Williamson, Duje Buric, Véronique Bourgarel, Laetitia Padovani, Steven C. Clifford, Olivier Ayrault, Eddy Pasquier, Nicolas André, Manon Carré

**Affiliations:** a Centre de Recherche en Cancérologie de Marseille (CRCM), Aix-Marseille Université, CNRS, Inserm, Institut Paoli Calmettes, Marseille, France; b Institut Curie, Inserm, CNRS, Université Paris-Saclay, Orsay, France; c Plateforme ICEP (CRCM), Aix-Marseille Université, CNRS, Inserm, Institut Paoli Calmettes, Marseille, France; d Service d'anatomie pathologique et de neuropathologie, Hôpital de la Timone, Assistance Publique-Hôpitaux de Marseille (APHM), Marseille, France; e Plateforme GenomEast, Institut de Génétique et de Biologie Moléculaire et Cellulaire (IGBMC), CNRS, Inserm, Université de Strasbourg, Illkirch-Graffenstaden, France; f Wolfson Childhood Cancer Research Centre, Newcastle University Centre for Cancer, Newcastle upon Tyne, United Kingdom; g Institut Méditerranéen de Biodiversité et d’Écologie marine et continentale (IMBE), Aix-Marseille Université, CNRS, IRD, Avignon Université, Marseille, France; h Service de Radiothérapie, Timone Hospital, Assistance Publique-Hopitaux de Marseille (AP-HM), Marseille, France; i Metronomics Global Health Initiative, Marseille, France; j Service d'Hématologie & Oncologie Pédiatrique, Timone Hospital, AP-HM, Marseille, France

**Keywords:** Beta-blockers, Bioenergetics, Medulloblastoma, Radiotherapy, Therapeutic combinations

## Abstract

**Background:**

Medulloblastoma is the most frequent brain malignancy of childhood. The current multimodal treatment comes at the expense of serious and often long-lasting side effects. Drug repurposing is a strategy to fast-track anti-cancer therapy with low toxicity. Here, we showed the ability of β-blockers to potentiate radiotherapy in medulloblastoma with bad prognosis.

**Methods:**

Medulloblastoma cell lines, patient-derived xenograft cells, 3D spheroids and an innovative cerebellar organotypic model were used to identify synergistic interactions between β-blockers and ionising radiations. Gene expression profiles of β-adrenergic receptors were analysed in medulloblastoma samples from 240 patients. Signaling pathways were explored by RT-qPCR, RNA interference, western blotting and RNA sequencing. Medulloblastoma cell bioenergetics were evaluated by measuring the oxygen consumption rate, the extracellular acidification rate and superoxide production.

**Findings:**

Low concentrations of β-blockers significantly potentiated clinically relevant radiation protocols. Although patient biopsies showed detectable expression of β-adrenergic receptors, the ability of the repurposed drugs to potentiate ionising radiations did not result from the inhibition of the canonical signaling pathway. We highlighted that the efficacy of the combinatorial treatment relied on a metabolic catastrophe that deprives medulloblastoma cells of their adaptive bioenergetics capacities. This led to an overproduction of superoxide radicals and ultimately to an increase in ionising radiations-mediated DNA damages.

**Interpretation:**

These data provide the evidence of the efficacy of β-blockers as potentiators of radiotherapy in medulloblastoma, which may help improve the treatment and quality of life of children with high-risk brain tumours.

**Funding:**

This study was funded by institutional grants and charities.

**Research in context**

**Evidence before this study**

In young patients with medulloblastoma, the current multimodal treatment allows 70% of children to survive up to 5 years but is often accompanied by serious and long-lasting side effects. Drug repositioning represents an attractive strategy to fast-track the development of new low-toxicity therapeutic options. Preclinical and clinical studies have shown that β-blockers can increase the efficacy of chemotherapy in drug-refractory cancers, including paediatric tumours such as neuroblastoma. However, far less is known about the ability of β-blockers to potentiate radiotherapy, which has never been studied in paediatric cancers. Moreover, the literature is divided regarding the mechanisms responsible for the anti-tumour properties of β-blockers. Here, we propose to analyse the combination of β-blockers with radiotherapy in models of high-risk medulloblastoma.

**Added value of this study**

We provide the evidence that low concentrations of propranolol, carvedilol and nebivolol improve the efficacy of ionising radiation in medulloblastoma cell lines, patient-derived tumour cells and spheroid micromasses, including those poorly responsive to radiation. In response to the ever-increasing need to find alternatives to animal experimentation, we have developed an innovative organotypic cerebellum model that confirmed the benefits of the combinatorial treatment. Although we showed that patient medulloblastoma biopsies exhibit detectable expression of β-adrenergic receptors, the efficacy of β-blockers in medulloblastoma cells does not result from the inhibition of the canonical targets but is instead driven by a rapid disruption of the mitochondrial bioenergetics. This leads to a sustained accumulation of superoxide radicals that potentiate the DNA damages caused by ionising radiation.

**Implications of all the available evidence**

Given the few druggable molecular targets identified in high-risk medulloblastoma and the fact that young age of patients limits treatment options, our work proposes an alternative approach in which drug repurposing could be quickly translated to the clinic to improve the efficacy of radiotherapy. In addition, as the dose of ionising radiations can be significantly reduced by adding β-blockers, this may help limit treatment long-term side effects and improve the quality of life of children with medulloblastoma. Lastly, our work highlights the interest of exploiting the ability of selected repositioned drugs to inhibit mitochondrial bioenergetics to design new therapeutic combinations with radiotherapy.

Alt-text: Unlabelled box

## Introduction

Medulloblastomas (MB) are embryonal tumours of the cerebellum and the most common malignant brain tumours of childhood. They have been classified into four main subtypes. WNT MB, has the most favorable clinical prognosis but accounts for only 10% of cases. SHH MB, and the other Non-WNT/Non-SHH subgroups (Group 3 and Group 4) are somewhat more aggressive and more frequently metastatic, with a poorer prognosis.^1^, ^2^, ^3^ The current multimodal treatment combines surgery, radiotherapy and chemotherapy.^4^^,^^5^ Overall, long-term survival is now achieved in 60-75% of patients but it comes at the expense of serious and often long-lasting side effects that can reduce independence and significantly alter the quality of life of survivors.^6^^,^^7^ High-risk MB are treated with radiation therapy with a cumulative dose of 54 Gy for irradiation of the posterior cerebellar fossa and additional 36 Gy for craniospinal irradiation. Although these doses are not always sufficient to control tumour progression, they cannot be increased as both acute toxicities and the cognitive and endocrinological sequelae would be too important in the long-term.^7^^,^^8^ Since these sequelae are even greater in young patients, radiotherapy is contraindicated in children under 3-5 years of age depending on countries.^9^^,^^10^ Therefore, new treatment options for MB patients are needed to improve the response to radiotherapy, with the aim of increasing the therapeutic benefits of ionising radiation (IR) and/or reducing its doses and associated deleterious side effects while maintaining its efficacy.

Drug repurposing consists in using already-approved drugs for indications that differ from those for which the drugs were originally developed. Toxicity and pharmacokinetic profiles are well documented, so that repurposed drugs can directly enter Phase II clinical trials. By reducing the time, expenses and risks associated with the development process, drug repurposing is an attractive strategy in anticancer therapeutics.^11^, ^12^, ^13^

One of the promising pharmacological classes to be repurposed is the β-adrenergic antagonists, or β-blockers. They are widely known for their regulatory properties in cardiovascular dysfunctions.^14^ To date, the use of propranolol for the treatment of severe hemangiomas of infancy represents one of the most successful examples of drug repurposing, with higher efficacy and fewer toxic side effects than the previous standard of care.^15^ Since then, our preclinical and clinical studies have shown that β-blockers can increase the efficacy of chemotherapy in drug-refractory cancers,^16^^,^^17^ including paediatric tumours such as neuroblastoma.^18^ β-blockers can impair fundamental biologic processes underlying tumour progression, such as cell proliferation, migration, tumour angiogenesis and metastasis. β-blockers have also been shown to sustain the response of irradiated gastric adenocarcinoma, colon adenocarcinoma or non-small cell lung cancer (NSCLC) *in vivo*,^19^, ^20^, ^21^, ^22^, ^23^ and improve survival outcomes in adult patients with intracranial meningiomas and NSCLC.^24^^,^^25^ These recent examples provide a strong rationale to combine β-blockers with radiotherapy in paediatric solid tumours, where this type of combination has never been evaluated. Here, we provide evidence that β-blockers can improve the efficacy of IR in MB cell lines and PDX-derived cells, by disrupting mitochondrial bioenergetics, independently of the β-adrenergic receptors.

## Material and methods

### Cell culture

The human MB cell lines DAOY (RRID: CVCL_1167), D341 Med (RRID: CVCL_0018) and D283 Med (RRID: CVCL_1155) were obtained from the ATCC biobank. HDMB-03 cells (RRID: CVCL_S506), were kindly provided by Sonia Martial and Gilles Pagès from the Institute for Research on Cancer and Aging (Nice, France). The human ONS-76 (RRID: CVCL_1624) and UW228-2 (RRID: CVCL_0572) cell lines were kindly provided by Janet Lindsey and Steven Clifford from the Wolfson Childhood Cancer Research Center (New Castle, UK). DAOY and UW228-2 cells are representatives of the WNT group; HD-MB03, D341 Med, D283 Med and ONS-76 cells are representatives of non-WNT/non-SHH MB.

All the cells were maintained at 37°C and 5% CO_2_. DAOY and D283 Med cells were grown in MEMα medium (Gibco, ref. 12561056) supplemented with 10% fetal calf serum (FCS; Gibco, ref. 26140079) and of 1% penicillin-streptomycin (PS; Gibco, ref. 15140122). HDMB-03 cells were maintained in RPMI 1640 medium (Gibco, ref. 21875034) supplemented with 10% FCS, 1% Non-Essential Amino Acids (Gibco, ref. 11140035) and of 1% PS. ONS-76 cells were grown in RPMI 1640 medium supplemented with 10% FCS and 1% PS. UW228-2 cells were grown in DMEM/F12 medium (Gibco, ref. 11320033) supplemented with 10% FCS and 1% PS. D341 Med cells were maintained in MEMα medium supplemented with 20% FCS and 1% PS.

A mtDsRed plasmid has been transfected in each cell line using Lipofectamine 2000 (Invitrogen, ref. 11668019) following the manufacturer's protocol. Stable transfectants were obtained after geneticin selection (0·8 mg/mL, Gibco, ref. 10131035) and two cycles of fluorescence-activated cell sorting (FACS). To establish β-blocker resistant cell lines, ONS-76 cells were exposed to increasing doses of propranolol (from 10 to 200 µM), carvedilol or nebivolol (from 2·5 to 20 µM), over 3 to 4 months. The resistant cell lines were named: ONS-76 RP, ONS-76 RC and ONS-76 RN, respectively, and were maintained in the same culture conditions as the parental ONS-76 cells (*i.e*., ONS-76 WT).

The murine SHH MB cell lines were obtained from spontaneous medulloblastoma arising from *Patched1^+/-^ C57BL/6* mouse model (RRID: MGI:2159769), as previously described.^26^

All the cells were tested for the absence of mycoplasma contamination (MycoAlert™, ref. #LT07-418, Lonza) at least once a month.

### Patient-derived xenografts culture

Patient-derived xenografts (PDXs) were generated from primary human MB samples and were maintained into the subscapular fat pad of Nude mice (RRID:MGI:5649750) as previously described.^27^ G3-PDX3, G3-PDX7 and SHH-PDX12 correspond to group 3 ICN-MB-PDX-3, group 3 ICN-MB-PDX-7 and SHH ICN-MB-PDX-12, respectively. For *in vitro* cultures, tumour cells were purified from the PDX using an enzymatic dissociation followed by a Percoll density gradient separation and cultured as previously described.^26^

### Drugs and reagents

The β-blockers were resuspended in dimethyl sulfoxide (DMSO). Stock solutions were stored at -20°C for Nebivolol (50 mM, Sigma-Aldrich, ref. #N1915) and Carvedilol (50 mM, Sigma-Aldrich, ref. #C3993) and at -80°C for Propranolol (150 mM, Selleckchem, ref. S4076). The antioxidants Mito-TEMPO (MT) and Troxerutin (TROX) were resuspended in DMSO and stock at -20°C. The solutions are diluted in culture medium ex-temporaneously for the experiments.

### Irradiation of MB cells, spheroids and organotypic cultures

Exposure to IR of the different culture models was performed in the Radiotherapy Department of Pr. Cowen (Timone Hospital, AP-HM, France). Water-equivalent RW3 phantom with a chamber adaptation plate was used for therapy dosimetry. Cells, spheroids and organotypic cultures were exposed to doses ranging from 1·8 Gy to 10 Gy, using the Synergy MLCi Elekta® linear accelerator with a beam of 6 MV and a flow rate of 400 UM/min. The PDX cells were irradiated in the RadExp platform of the Curie Institute on the X-Rad 320 equipment (Precision X-ray irradiation).

### Cell growth and survival assays

Cell viability assays were performed as previously described.^18^ Briefly, the human MB cells were seeded in flat bottom 96-well microplates (2,000 cells/well for DAOY, ONS-76 and UW228-2; 9,000 cells/well for HD-MB03 and D283 Med; 12,000 cells/well for D341 Med) for 24 h. Cells were then exposed to β-blockers alone or in combination with IR for 72 h. Metabolic activity was detected by addition of Alamar Blue and spectrofluorimetric analysis using a PHERAstar® FS multi-Plate Reader (BMG LABTECH; λ_ex_ 540 nm / λ_em_ 590 nm). IC_50_ values were determined as previously described.^28^

For IncuCyte experiments (RRID:SCR_019874), SHH MB tumour cells were plated in 96-well plates (5,000 cells/well for murine SHH-MB and 7,500 cells/well for ICN-MB-PDX-12), pre-coated with poly-D-lysine (EMD Millipore, ref. A-003-E) and Matrigel (BD Biosciences, ref. 354234). The next day, tumour cells were treated with a range of concentrations of β-blockers or the control, as indicated in figures. Propidium iodide (PI, Sigma Aldrich; 0·3 μg/ml) was also added to the medium to evaluate cell death. Then, the plates were scanned for phase contrast and PI staining during 72-96 h, using the IncuCyte® live cell analysis system with a 4X objective. Proliferation was measured using quantitative kinetic processing metrics from time-lapse image acquisition and showed as percentage of culture confluence over time. For the PI staining, the percentage of PI positive cells was divided by the percentage of cell confluence for each well, thus indicating the level of dead cells in each well.

For the CellTiter-Glo® Luminescent Cell Viability Assay, Group 3 MB tumour cells were cultured in neurospheres in round bottom 96-well plates (5,000 cells/well). Tumour cells were then treated either 1) once with a range of concentrations of β-blockers or 2) daily with β-blockers and/or IR for five consecutive days. Then, the cell viability was evaluated 72 h later using the CellTiter-Glo® Luminescent Cell Viability Assay according to the manufacturer's instructions (Promega Corporation, ref. G7570).

### Spheroid growth assay

DsRed-expressing MB cells were plated in round bottom 96-well microplates (1,200 cells/well for HD-MB03 – 1,500cells/well for UW228-2 and DAOY – 2,000 cells/well for ONS-76, D283 Med and D341 Med) in a culture medium containing 10% FBS and 20% methyl cellulose (Sigma-Aldrich, ref. M7027) for 72 h. Spheroids were then daily treated with β-blockers and/or IR for five days. Spheroid growth was quantified over time by acquisition of DsRed fluorescence signal using the PHERAstar® FS multi-plate reader (λ_ex_ 580 nm/λ_em_ 620 nm – “well scanning” 10 × 10). Images were captured with the JuLI™Stage live imaging system (NanoEntek).

### Cerebellar organotypic model development and analysis

To establish organotypic cultures of cerebellar tissues, mouse cerebellums were surgically harvested and sectioned into 250 µm thick slices using a vibrating blade microtome (RRID:SCR_016495). A spheroid formed from DsRed-expressing MB cells was then grafted onto each cerebellum slice. These organotypic co-culture models were then placed on inserts with 0,4 µm pore size membranes (Falcon®, ref. 353090) and maintained in medium containing 50% MEMα, 25% horse serum (Gibco, ref. 16050122), 25% Hanks' Balanced Salt Solution (HBSS; Gibco, ref. 14065056), 10 mM HEPES buffer (Gibco, ref. 15630106), 28 mM Glucose (Gibco, ref. 15023021), 1% L-Glutamine (Gibco, ref. 25030081) and 1% PS. After daily exposure to IR and/or β-blockers for 5 consecutive days, tumour growth and invasion within the cerebellum slices were analysed over time, using the JuLI™ Stage imaging system and the PHERAstar® FS multi-plate reader (λ_ex_ 580 nm/λ_em_ 620 nm - fluorescence signal acquisition with a 15 × 15 matrix scanning mode).

### Sample preparation and immunohistochemistry

Samples were fixed overnight at 4°C with 4% formaldehyde and prepared for paraffin inclusion using automated tissue processor ASP 300 (RRID:SCR_018916). Dehydration, clarification, and infiltration steps were performed by successive absolute ethanol, histolemon and paraffin baths. After FFPE-embedding, samples were cut at 3µm-thickness with HM340E microtome (Thermo Scientific). Hematoxylin Eosin Safran staining was performed using automated H&E staining Dako CoverStainer.

Ki-67- and γH2AX-immunohistochemistry was carried out with rabbit anti-Ki67 antibody (RRID:AB_443209) and with mouse anti-γH2AX antibody (Merck Milliopore, ref. JBW301) on a Ventana Discovery XT (RRID:SCR_018643). After deparaffinisation, antigen retrieval was performed with Citrate-based buffer pH 6·5 (RiboCC Solution, CC2, ref. 760-107). The primary antibodies were incubated for 20 min at 37°C then an OmniMap anti-Rabbit HRP Detection Kit (ref. 760-149) was used with DAB. Finally, the counterstaining was done with hematoxylin and slides were cleaned, dehydrated and coversliped with permanent mounting media. The microscopic analysis of the tissues was carried out by the pathologists of the Neuropathology Department (Timone Hospital, AP-HM, France).

### Western-blot

Cells were lysed in RIPA buffer (50 mM Tris-HCl (pH 8), 150 mM NaCl, 1% Triton X-100, 0·1% SDS) to which was extemporaneously added a cocktail of phosphatase and protease inhibitors (Sigma-Aldrich, ref. PPC1010). Protein concentration was determined using a Protein Assay Dye Reagent Concentrate (Bio-Rad, ref. #5000006EDU) according to the Bradford method. Proteins (30 µg) were separated by polyacrylamide-SDS gel (10% Mini-PROTEAN® TGX™ Precast Gels, Bio-Rad, ref. 4561034) and were transferred on nitrocellulose membrane (GE Healthcare Life Sciences). Primary antibodies used were directed against COX2, β-actin or γH2AX (RRID: AB_2571729, RRID: AB_2242334 and RRID: AB_2799949, respectively). Secondary antibody coupled to peroxidase (RRID: AB_2099233 for anti-rabbit, or RRID: AB_330924 for anti-mouse IgG antibody) were used for revelation, performed using the Luminata chemiluminescence detection kit (Millipore, ref. WBLUC0100). Images were acquired using the G-BOX phosphoimager (Ozyme) and signal quantification was realised by Image J® software (RRID:SCR_003070).

### Measurement of superoxide production

MB cells were seeded on 96-well microplates (2,000 cells/well for ONS-76 and 9000 cells/well for HD-MB03) for 24 h and exposed to IR and/or β-blockers for 6 h. 3D spheroids of MB cells were formed 3 days before treatment, and exposed to IR and/or propranolol for 6 h. Superoxide anion production was assessed by adding 10% V/V of WST-1 reagent (Roche, ref. 11644807001) in the wells for 30 min at 37°C. Absorbance was measured at 450 nm with a PHERAstar® FS multi-plate reader. To normalise superoxide production to the cell number in each condition, cells were fixed with 1% glutaraldehyde and stained with a solution of 1% (W/V) crystal-violet in 20% methanol (Sigma-Aldrich). The dye has finally been solubilised in DMSO to measure absorbance at 600 nm.

### Colony formation assay

Ninety-six-well microplates were coated with 1% agarose for 24 h. Two hundred and fifty ONS-76 cells and 500 HD-MB03 cells per well were then plated in a 10% Matrigel®- containing medium (Corning, ref. 354234) for 24 h and exposed to β-blockers and/or IR. Photos of the colonies were captured with the JuLI™ Stage imaging system and quantified using the Image J® software, 7 and 10 days after treatment initiation for ONS-76 and HD-MB03 cells, respectively.

### Analysis of energy metabolism

Energy metabolism analysis was performed using the Seahorse XFe24® extracellular flux analyser (RRID:SCR_019539). Adherent MB cells were seeded in XF24 V7-PS plates (10,000 cells/well for DAOY; 12,000 cells/well for ONS-76 and UW228-2; 30,000 cells/well for HD-MB03; Agilent, ref. 102340-100) for 24 h. Cells were then exposed to β-blockers and/or IR for 6 or 24 h. Oxygen consumption rate (OCR) was measured using XF cell Mito Stress test (Agilent, ref. 103015-100). One hour before measurement, culture medium was changed with unbuffered DMEM, 143 mM NaCl, 2 mM glutamine, 1 mM sodium pyruvate and 10 mM glucose. OCR was measured after injection of 1 µM Oligomycin, 0·5-1 µM FCCP and 0·5 µM antimycin A/rotenone mixture. The acidification rate of the extracellular environment (ECAR) was measured using XF Glycolysis stress test (Agilent, ref. 103020-100). Cell culture medium was replaced with unbuffered DMEM, 143 mM NaCl, 2 mM glutamine and 1 mM sodium pyruvate. ECAR was measured after the addition of glycolysis modulators: 10 mM glucose, 1 µM oligomycin and 100 mM 2-deoxy-glucose.

Maximal mitochondrial respiration was measured after injection of FFCP. OCR-linked ATP production was calculated with difference between basal and maximal respiration values, while glycolytic reserve was calculated as the difference between oligomycin-enhanced and glucose-mediated ECAR values.

To normalise the data to cell number, cells were fixed with glutaraldehyde 1%, stained with violet crystal in 20% methanol (Sigma-Aldrich) and solubilised with DMSO to measure absorbance at 600 nm with PHERAstar®FS multi-plate reader. A calibration range established for each type of cell was finally used to convert the absorbance values into cell numbers.

### Quantitative RT-PCR analysis

The expression of β1-, β2- and β3-adrenergic receptor genes (*ADRB1, ADRB2* and *ADRB3*) was examined using real-time quantitative RT-PCR. Total cellular RNA was extracted from MB cells using the RNeasy Mini Kit according to the protocol supplied by the manufacturer (Qiagen, ref. 74104). RNA concentration was measured with the NanoVue™ Plus spectrophotometer (GE Healthcare Life Sciences). Reverse transcription of RNAs was done using QuantiTect® Reverse Transcription kit (Qiagen, ref. 205311) and real-time PCR was ran using the QuantiTect® SYBR® Green PCR kit (Qiagen, ref. 204143) on a LightCycler®480 Instrument (RRID:SCR_020502). The primers were synthetised by Qiagen (QuantiTect® Primer Assay, ref. 249900) according to the sequences described in Cao DX *et al*.^29^ Gene expression level of *ADRB1, ADRB2* and *ADRB3* was determined using 2^-∆Ct or 2^-∆∆Ct method, normalised to *GAPDH* as housekeeping gene.

### siRNA transfection

Small interfering RNAs directed against β1- or β2- adrenergic receptors (Silencer Select siRNA, ref. #4392420 - S1118, S1119 and S1120 for *ADRB1* and S1121, S1122 and S1123 for *ADRB2*, ThermoFischer), and non-targeted SignalSilence® Control siRNA (Cell signaling, ref. #6568S) were transfected in MB cells using Lipofectamine™ 3000 (Invitrogen, ref. L3000015) according to the protocol supplied by the manufacturer. Verification of the successful transfection was performed by quantitative RT-PCR, as described above.

### RNA sequencing

The MB cells ONS-76 WT, ONS-76 RP, ONS-76 RC and ONS-76 RN were homogenised using a Buffer RLT (Qiagen, ref. 79216) and DNA-free cell lysates were obtained using genomic DNA purification columns (Qiagen). Extraction of total RNA was performed using the RNeasy Plus Mini kit (Qiagen, ref: 74134), according to the protocol supplied by the manufacturer. RNA was quantified using a NanoVue™ Plus spectrophotometer (GE Healthcare Life Sciences). RNA-Seq libraries were generated from 600 ng of total RNA using TruSeq Stranded mRNA Library Prep Kit and TruSeq RNA Single Indexes kits A and B (Illumina), as previously described.^30^ The final cDNA libraries were checked for quality and quantified using capillary electrophoresis. Libraries were then sequenced on an Illumina HiSeq4000 sequencer (RRID:SCR_016386) as single end 1 × 50 base reads. Image analysis and base calling were performed using RTA 2.7.3 and bcl2fastq 2.17.1.14. Reads were preprocessed using Cutadapt v1.10^31^ in order to remove adapter, polyA and low-quality sequences (Phred quality score below 20), reads shorter than 40 bases were discarded for further analysis. Reads mapping to rRNA were also discarded (this mapping was performed using Bowtie v2.2.8.^32^ Reads were then mapped onto the hg38 assembly of human genome using STAR v2.5.3a.^33^ Gene expression was quantified using htseq-count v0.6.1p1^34^ and gene annotations from Ensembl release 99. Statistical analysis was performed using R 3.3.2 and DESeq2 1.16.1 Bioconductor library.^35^

Read counts for *ADRB1, ADRB2* and *ADRB3* expression in primary MB samples from patients were produced by aligning paired end RNA-seq (∼90 M read/sample Illumina HiSeq2500; RRID:SCR_020123) reads to HG19 genome using STAR-align.^33^ Read counts were produced using HT-SEQ-count. DESeq 2 (R/Bioconductor) was used to normalise reads to library size and variance stabilised data (VSD) was generated using the vsd function. Statistical testing for differential expression across groups was performed using an ANOVA test.

### Ethics

Tumour samples from individuals with confirmed medulloblastoma diagnosis were used for RNA-seq analysis. These were provided as part of UK CCLG-approved biological study BS-2007-04 and/or with approval from Newcastle North Tyneside Research Ethics Committee (study reference 07/Q0905/71); informed, written consent was obtained from parents of all patients younger than 16 years.

All animals for PDX were housed in the animal facility of the Institut Curie, in accordance with the recommendations of the European Community (2010/63/UE) for the care and use of laboratory animals. Experimental procedures were specifically approved by the ethics committee of the Curie Institute CEEA-IC #118 (approval number: 03130.02, C91471108 and Authorisation *APAFiS# 26879-2020081315161665-v1* given by National Authority) in compliance with the international guidelines. Cerebellar explants were obtained from the animal facility of the Faculty of Pharmacy, in accordance with the recommendations of the European Community (approval number: E 13 055 20).

### Statistics

All experiments were performed in independent replicates and statistical significance was determined by ANOVA or student's t test using the GraphPad Prism 6 software (RRID:SCR_002798). A significant difference between two conditions is defined as: * *p* < 0·05, ** *p* < 0·005, *** *p* < 0·001.

### Role of funders

The study sponsors did not have any role in study design, in the collection, analysis, interpretation of data, in the writing of the manuscript or in the decision to submit it for publication.

## Results

### β-blockers inhibit the proliferation and survival of MB cell lines and patient-derived tumour cells

**Figure 1:**
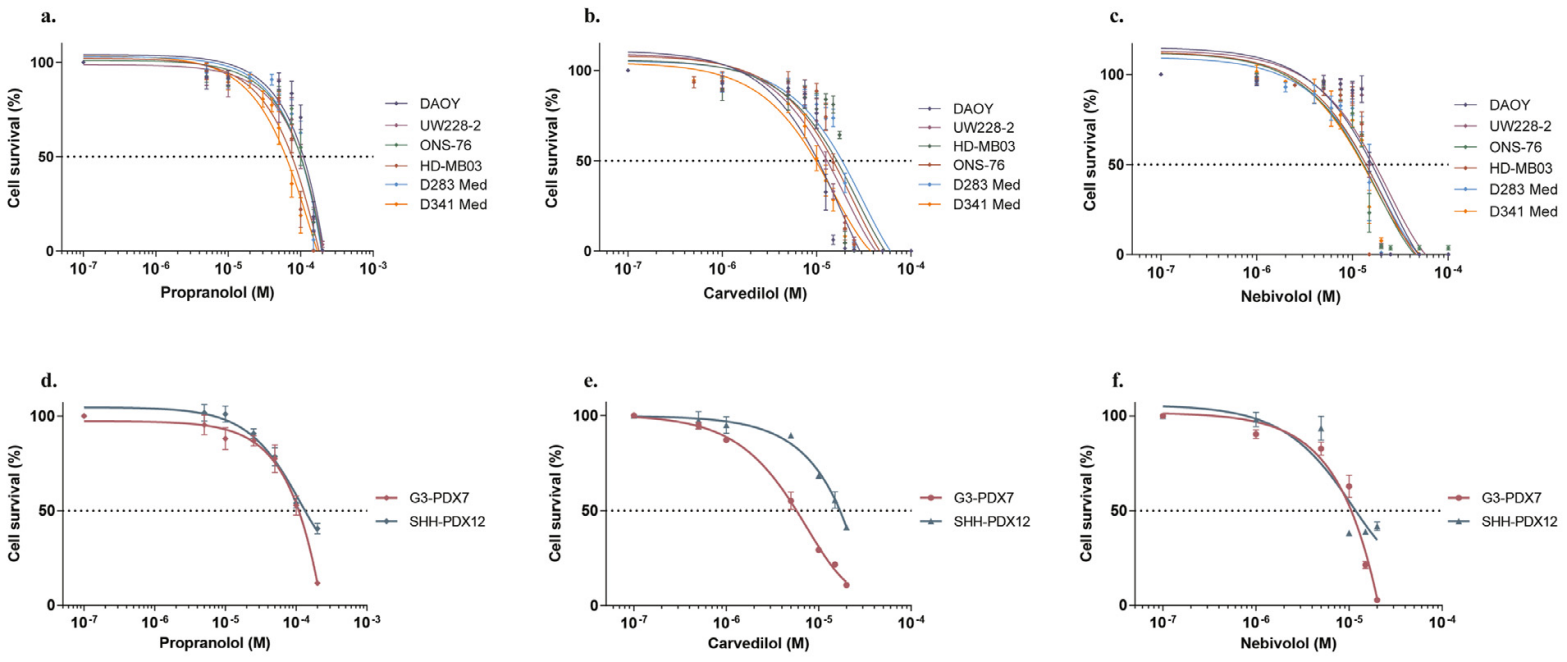
**Inhibition of MB cell survival by β-blockers**. Cell survival analysis in six human MB cell lines by Alamar Blue assay after 72 h of treatment with increase concentrations of propranolol **(a)**, carvedilol **(b)** and nebivolol **(c)**. Values are the average of ten independent experiments ± standard deviation (SD), with a biological triplicate in each experiment. Evaluation of cell survival using CellTiter-Glo® assay in neurosphere cultures of human MB cells from G3-PDX7 and IncuCyte® live cell analysis system for SHH-PDX12 cells, after 96 h of treatment with increase concentrations of propranolol **(d)**, carvedilol **(e)** and nebivolol **(f)**. Values are the average of three independent experiments ± SD, with a biological triplicate in each experiment. *p < 0·05; **p < 0·005; ***p < 0·001.

To determine the anti-proliferative properties of three different β-blockers with different selectivity profiles for adrenergic receptors – *i.e.,* non-selective β-blocker propranolol, mixed α/β-blocker carvedilol and β1-selective antagonist nebivolol –, we first used a panel of six human MB cell lines characteristic of group 2 SHH (UW228-2 and DAOY) and non-WNT/non-SHH group (HD-MB03, ONS-76, D283 Med and D341 Med) tumours. All tested β-blockers inhibited the proliferation of MB cells, irrespective of their group (Figures 1a-c), with IC_50_ values ranging from 60-120 µM for propranolol, 12-15 µM for carvedilol and 13-15 µM for nebivolol (Table 1). We further showed that the activity of β-blockers results from both the inhibition of cell growth and the induction of cell death in murine SHH-MB cells (Figure S1a-f). To evaluate the three β-blockers in more clinically relevant cellular models, we cultured primary cells from group 3 and SHH patient-derived xenografts MB tumours (G3-PDX7 and SHH-PDX12, respectively). We confirmed the dose-dependent efficacy of propranolol, carvedilol and nebivolol in inhibiting cell survival of these PDX-derived cells (Figures 1d-f), as well as their ability to inhibit cell proliferation and induce cell death (Figure S1g-l).

**Table 1:** IC_50_ values of propranolol, carvedilol and nebivolol after 72 h of treatment of non-Wnt human MB cells (DAOY, UW228-2, HD-MB03, ONS-76, D283 Med, D341 Med), mice MB cells (murine SHH-MB) and PDX-isolated cells (G3-PDX7 and SHH-PDX12), determined by GraphPad Prism software. Values are the average of at least three independent experiments ± SD.

	Propranolol	Carvedilol	Nebivolol
**DAOY**	124.9 ± 3.2	14.9 ± 0.1	15.0 ± 0.1
**UW288-2**	101.5 ± 3.8	14.6 ± 0.1	14.5 ± 0.1
**HD-MB03**	91.1 ± 3.1	12.9 ± 0.4	12.9 ± 0.4
**ONS-76**	111.8 ± 2.3	13.2 ± 0.2	13.2 ± 0.2
**D283 Med**	105.3 ± 2.7	13.9 ± 0.4	13.9 ± 0.4
**D341 Med**	62.0 ± 1.4	13.0 ± 0.3	13.0 ± 0.3
**murine SHH-MB**	31.2 ± 2.7	6.3 ± 0.4	1.7 ± 0.3
**G3-PDX7**	93.2 ± 6.0	5.3 ± 0.2	10.9 ± 0.4
**SHH-PDX12**	133.7 ± 8.2	17.2 ± 0.6	13.0 ± 1.1

### β-blockers enhance IR-mediated inhibition of MB cell proliferation and clonogenicity

**Figure 2:**
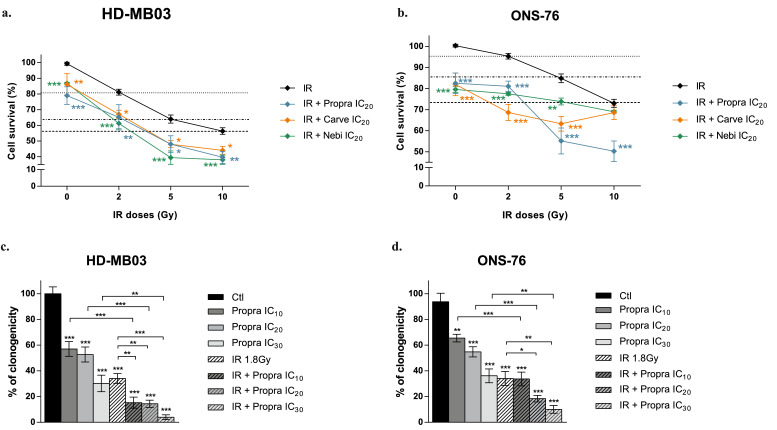
**Increase of IR efficacy by β-blockers in MB cells**. HD-MB03 **(a)** and ONS-76 **(b)** cell survival analysis by the Alamar Blue assay after 72 h of treatment with IC_20_ of propranolol (propra), carvedilol (carve) or nebivolol (nebi) alone and combined to radiotherapy (IR) 2, 5 and 10 Gy. Values are the average of three independent experiments ± standard error of mean (SEM). Quantification of HD-MB03 **(c)** and ONS-76 **(d)** cell colonies, using the ImageJ™ software, respectively after 10 or 7 days of treatment with increase concentrations of propranolol (propra) or radiotherapy (IR) 1·8 Gy alone and their combination. Values are the average of three independent experiments ± SEM. *p < 0·05; **p < 0·005; ***p < 0·001.

To study the combination between β-blockers and radiotherapy in MB cells, we first tested a single co-treatment of IR at 2, 5 or 10 Gy and low concentrations – IC_20_ – of propranolol, carvedilol or nebivolol. Results showed that the addition of the β-blockers led to a two-fold reduction in the dose of IR while maintaining the same activity in HD-MB03 cells (Figure 2a). For example, irradiation at 2 Gy combined with IC_20_ of propranolol is as effective in reducing cell survival as irradiation alone at 5 Gy. IR potentiation by propranolol was also found in ONS-76 cells (Figure 2b) and in the three other tested MB cell lines (Figure S2a-c). Similar effects were observed with low concentrations of carvedilol or nebivolol combined to IR in the different MB cell lines (Figures 2a-b and Figure S2a-c). To better explore the potential of these combinations in MB cell radiosensitivity, we conducted clonogenic assays. HD-MB03 and ONS-76 cells were exposed to propranolol and/or IR at 1·8 Gy, which is the daily radiation dose the most widely used in the clinic. As expected, the number of colonies was reduced by IR by 64 ± 5 % and 60 ± 5 % in HD-MB03 and ONS-76 cultures, respectively (Figures 2c-d). Our results also demonstrated that propranolol decreased the clonogenicity of MB cells, in a dose-dependent manner and significantly enhanced the efficacy of IR (Figures 2c-d and Figure S2d). For instance, the clonogenic capacity of HD-MB03 and ONS-76 cells exposed to the combination of IR with propranolol IC_20_ was reduced by 86 ± 3 % and 82 ± 3 %, respectively (Figures 2c-d, *p* < 0·001 *vs* control).

**Figure 3:**
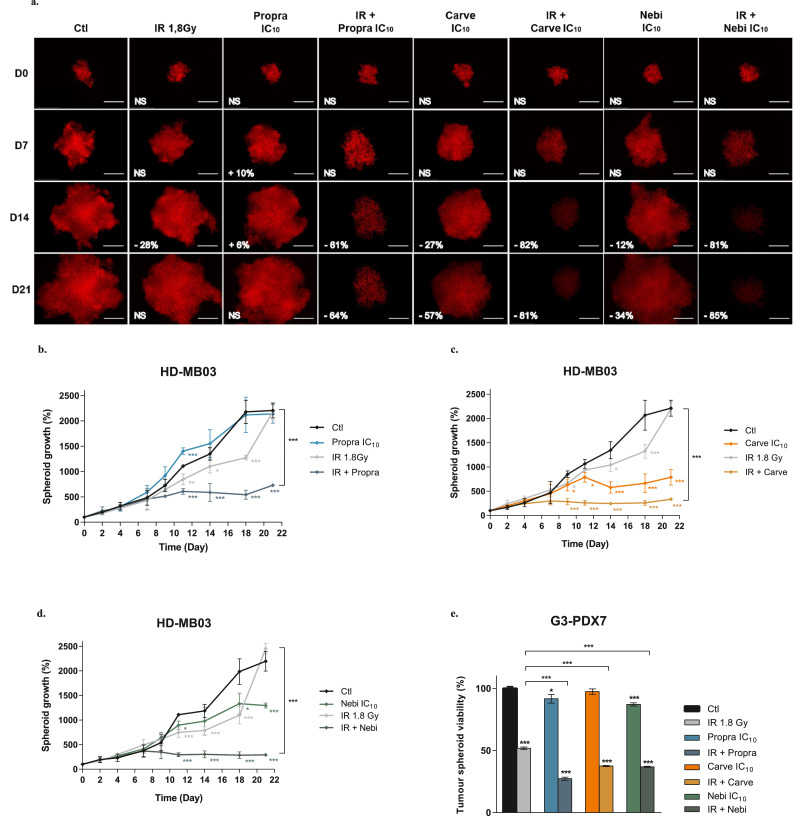
**β-blockers enhance IR activity in 3D spheroids of MB cell lines and MB PDX cells.** For five consecutive days, the 3D tumour micromasses were exposed to daily low doses (IC_10_) of β-blockers or radiotherapy (IR) 1·8 Gy alone and their combination. **(a)** Representative images of DsRed-expressing HD-MB03 spheroids, acquired over time with the JuLi™ Stage system. Treatment efficacy was expressed as a percentage of spheroid growth inhibition *vs* control spheroids (Ctl), determined by quantifying the DsRed signal with the PHERAstar microplate reader. Scale bars: 500 µm. HD-MB03 spheroid growth measured by acquisition of the DsRed signal over 21 days after treatment with IC_10_ of propranolol (propra) **(b)**, carvedilol (carve) **(c)** and nebivolol (nebi) **(d)** alone or combined with radiotherapy (IR) 1·8 Gy. Values are the average of n = 15 samples per condition, from at least four independent experiments ± standard deviation (SD). **(e)** Spheroid viability assessment by using the CellTiter-Glo® assay in G3-PDX7 cells after 72 h of treatment with IC_10_ of propranolol (propra), carvedilol (carve) and nebivolol (nebi) alone or combined with radiotherapy (IR) 1·8 Gy. Values are the average of n = 10 samples per condition, from three independent experiments ± SD. *p < 0·05; **p < 0·005; ***p < 0·001.

To confirm the interest of such a combination in 3D tumour micromasses, we developed tumour spheroid models from MB cells stably expressing DsRed. For five consecutive days, spheroids were exposed to daily low doses of β-blockers alone or in combination with IR at carvedilol and nebivolol sustainably potentiate IR in HD-MB03 spheroids, as compared with IR alone (Figures 3a-d). While IR did no longer significantly impact the spheroid growth at day 21 (2317 ± 60 % growth in irradiated *versus* 2206 ± 79 % growth in control spheroids; p > 0·05), the co-treatment with propranolol, carvedilol and nebivolol decreased the spheroid growth to 724 ± 5 %, 335 ± 2 % and 292 ± 5 %, respectively (p < 0·001, Figures 3a-d). Similar results were obtained in ONS-76 spheroids (Figure S3a). In addition, β-blockers were able to restore IR efficacy in UW228-2 and D283 Med spheroids that were unresponsive or minimally responsive (Figure S3b-c) and they could further increase IR efficacy against the highly radio-sensitive D341 Med spheroids (Figure S3d). Finally, to evaluate the efficacy of daily co-treatment on primary MB cells, we established 3D neurospheres from the G3-PDX7 cells. Our results confirmed that low concentrations of propranolol, carvedilol, or nebivolol highly potentiated the effects of IR (Figure 3e). The use of a second group 3 PDX model (G3-PDX3) further validated the relevance of combining β-blockers in co-treatment with daily radiotherapy in MB (Figure S3e). Altogether, our results demonstrated that β-blockers can improve the efficacy of IR in *in vitro* MB models.

### Fractionated IR is potentiated by daily low doses of β-blockers in cerebellar organotypic models

**Figure 4:**
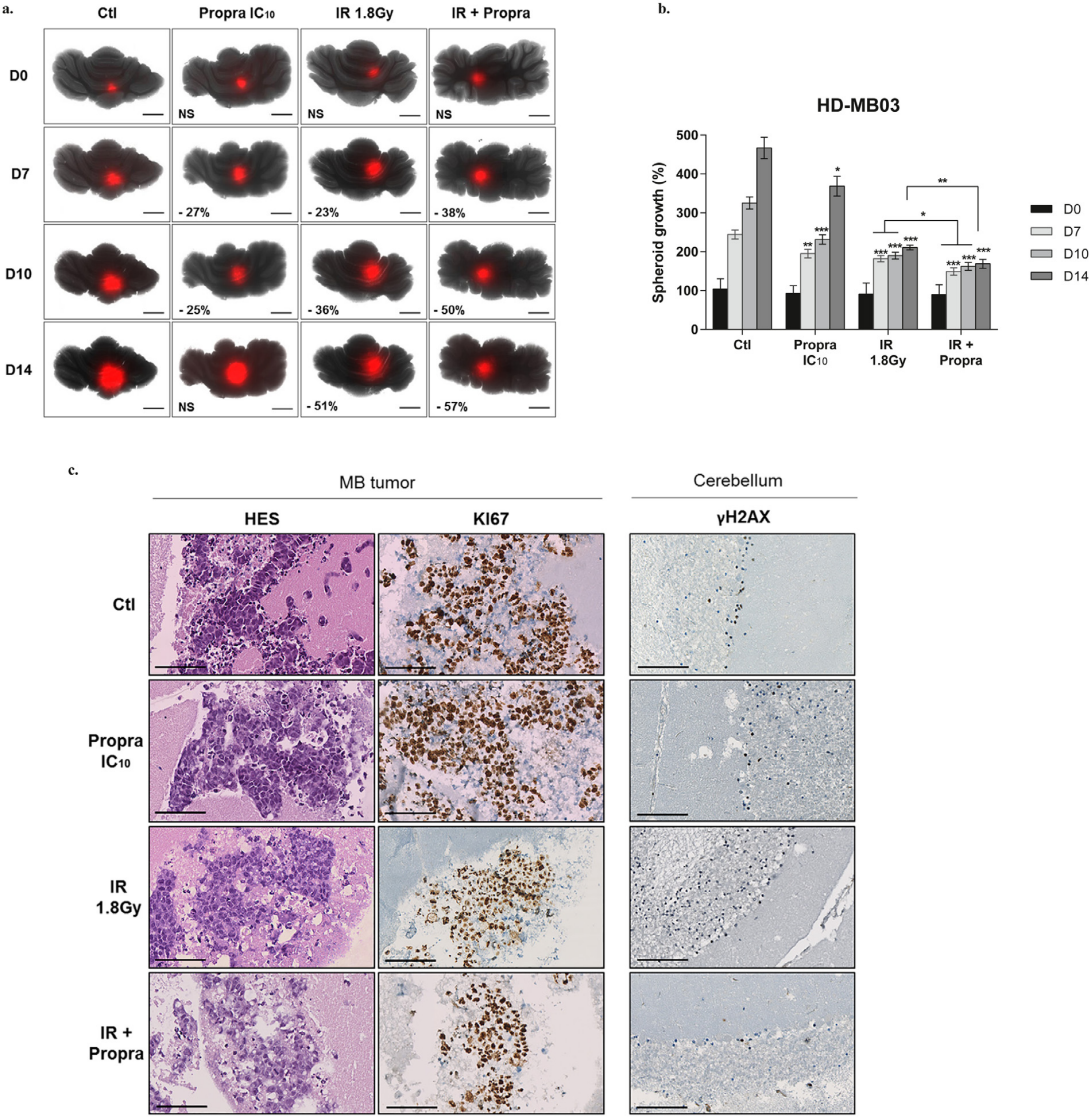
**Benefits of daily low concentrations of β-blockers combined to IR in *ex-vivo* MB organotypic model.** For five consecutive days, the organotypic cerebellar co-cultures were exposed to daily low doses (IC_10_) of propranolol alone or combined to radiotherapy (IR) 1·8 Gy. **(a)** Representative pictures, acquired with the JuLi™ Stage live imaging system, of DsRed-expressing HD-MB03 tumour micromasses grafted in slices of healthy cerebellum. Scale bars: 1 mm. Results were expressed as percentage of growth inhibition in treated *vs* control organotypic models (Ctl). **(b)** HD-MB03 tumour growth was measured over 14 days by acquisition of the DsRed signal with the PHERAstar microplate reader (well-scanning mode). Values are the average of n = 15 samples per condition, from five independent experiments ± standard deviation (SD). **(c)** Representative pictures of HES, KI67 and γH2AX immunostaining in organotypic models, control (Ctl) and treated with IC_10_ of propranolol (propra), radiotherapy (IR) 1·8 Gy or their combination. Scale bars: 100 µm. *p < 0·05; **p < 0·005; ***p < 0·001.

To evaluate the potential of the β-blockers and IR combination in more clinically relevant conditions, we developed an organotypic cerebellar model in which MB spheroids stably expressing DsRed were grafted into slices of healthy mouse cerebellum. These innovative cultures were daily exposed to IR (1·8 Gy) and/or very low concentrations of propranolol (IC_10_ *i.e.*, 25 µM) for five consecutive days. After seven days, our data showed that monotherapies reduced the growth of HD-MB03 tumour masses by 23 ± 5% and 27 ± 5% in organotypic models subjected to IR and propranolol, respectively (*p <* 0·001 and *p <* 0·05, respectively; Figures 4a-b). The combinatorial treatment resulted in a reduction of 38 ± 6% (*p <* 0·001 compared with control), which significantly increased the efficacy of IR (*p <* 0·001, Figures 4a-b). The potentiating effect persisted over time, with the combination reducing tumour growth by 57 ± 6% after 14 days (*p <* 0·001 *vs* control and *p <* 0·05 *vs* IR; Figures 4a-b). The benefits of combining propranolol with IR were also confirmed in organotypic cerebellar models transplanted with ONS-76 tumour masses (Figure S4a-b). After 14 days, the organotypic cultures were fixed, sectioned, and subjected to HES and Ki67 staining (Figure 4c). Microscopic analysis of these labelling patterns showed that the combinatorial treatment did not induce histological lesions in the non-tumour cerebellar tissue, including in the MB periphery. Furthermore, γH2AX staining of the organotypic models showed that the co-treatment with IR and propranolol did not induce DNA damage in the non-tumour tissue either (Figure 4c, Table 2, and positive control in FigureS4c). This suggests that the combination is effective in significantly reducing MB tumour mass without inducing additional damage to the cerebellum.

**Table 2:** yH2AX positive cells (% +/- SD) in the cerebellum of the organotypic models. Quantification was made by microscopic analysis in the Neuropathology Department. *p* values > 0·05 indicate no significant variations.

	%	SD	*p*	
**Control**	1.6	1.34	/	
**Propra IC10**	1.6	1.30	0.49	***vs* Control**
**IR 1,8 Gy**	2.0	1.00	0.30	***vs* Control**
**IR + Propra**	1.2	0.63	0.28	***vs* Control**
			0.08	**vs IR**

### β-blocker efficacy and potentiation of IR are independent of β-adrenergic receptors in MB cells

**Figure 5:**
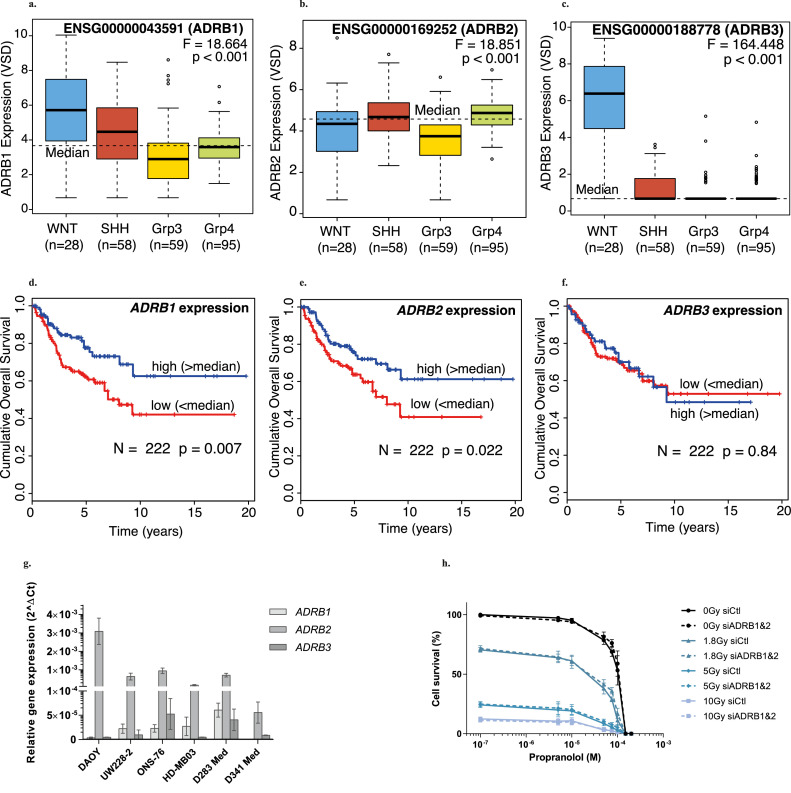
**β-Adrenergic receptors (β-AR) are not involved in the response of MB cells to β-blockers.** Boxplot showing expression of *ADRB1***(a)**, *ADRB2***(b)** and *ADRB3***(c)** by subgroup in MB patient cohort (n = 240). Kaplan-Meier curves reporting patient overall survival stratifying patients by > median and < media expression for *ADRB1***(d)**, *ADRB2***(e)** and *ADRB3***(f)** expression levels. **(g)** Relative gene expression of *ADRB1, ADRB2* and *ADRB3* quantified by qRT-PCR using *GAPDH* as housekeeping gene (n = 8 per condition, from four independent experiments). Calculation has been done by the 2^-∆Ct method. Values are the average of independent experiments ± standard error of mean (SEM). **(h)** Cell viability analysis by using the Alamar Blue assay after 72h of treatment with radiotherapy (IR) 1·8, 5 and 10 Gy and increase concentrations of propranolol in HD-MB03 cells transfected with siRNA control (Ctl) or siRNA *ADRB1&2*. Values are the average of three independent experiments ± SEM, with a biological triplicate in each experiment. *p < 0·05; **p < 0·005; ***p < 0·001.

The strong synergism between IR and β-blockers in MB stresses the need for a better understanding of the underlying mechanism(s). Since β-blockers antagonise the β-adrenergic receptors (β-ARs) in the cardiovascular system, we first evaluated the expression pattern of β-AR genes *ADRB1, ADRB2* and *ADRB3* in MB tumours from a cohort of 240 patients (Figures 5a-c). While there are significant differences in expression of β-AR isoforms across MB groups (each *p <* 0·001), the median expression of *ADRB2* is the highest, followed by *ADRB1*, and WNT MB are the only samples that express high levels of *ADRB3*. Kaplan Meier and Cox regression analyses revealed that high expression (>median) of *ADRB1* and *ADRB2* were associated with a good prognosis in a cohort of 222 patients (Figures 5d-f). We then quantified β-AR mRNA levels in the six human SHH and non-WNT/non-SHH group MB cell lines studied. Consistently with the results obtained with patient samples, *ADRB2* and *ADRB3* are the major and the minor isoforms across the panel of cell lines, respectively (Figure 5g). Interestingly, despite being as sensitive as the other cell lines to the β1-selective antagonist nebivolol (Figure 1c), neither the DAOY nor the D341 Med cell lines express *ADRB1* (Figure 5g). This suggests that the efficacy of β-blockers in MB cells may not rely on the canonical β-adrenergic pathway. To confirm this hypothesis, we silenced *ADRB1* and *ADRB2* in HD-MB03 and ONS-76 cells using RNAi technology (Figure S5a-b), β3-AR not being a target of any of the three β-blockers tested here. Our data showed that the efficacy of propranolol, carvedilol and nebivolol alone or in combination with IR was not impacted by β-AR silencing (dotted *vs.* solid lines, Figure 5h and Figure S5c-g). Moreover, β-AR siRNA did not improve the effects of IR alone in HD-MB03 cells, regardless of the dose used (Figure 5h and Figure S5c-d). In ONS-76 cells, β-AR silencing even significantly reduced the effects of IR (*p <* 0·05; Figure S5e-g). These results indicate that β-ARs are neither involved in β-blocker-induced cytotoxicity nor in radio-sensitisation of MB cells and support the idea that β-blockers trigger an alternative signaling pathway to potentiate radiotherapy in MB cells.

### Response to β-blockers is associated with inhibition of energy metabolism in MB cells

**Figure 6:**
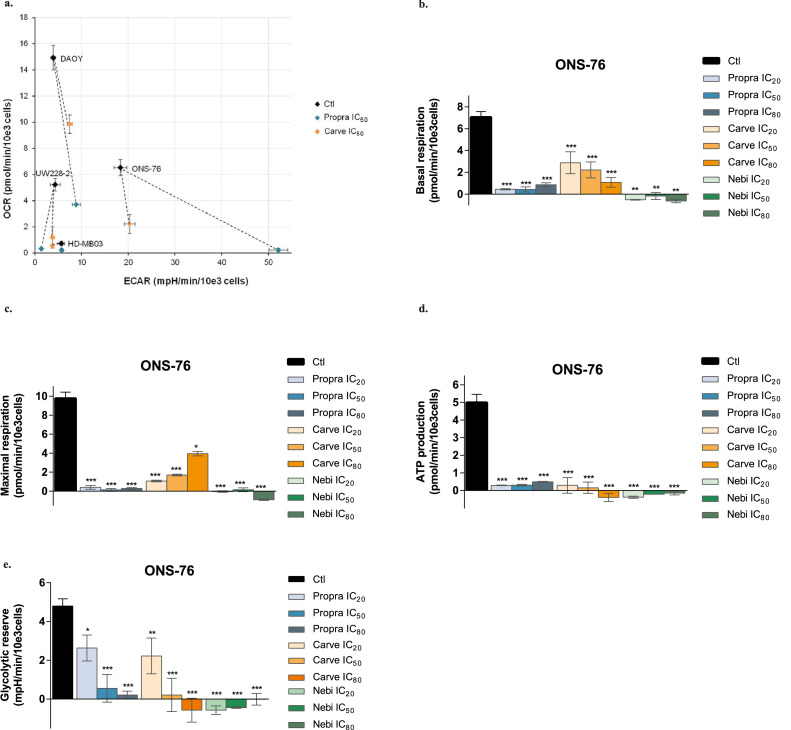
**β-blockers inhibit MB cell energy metabolism. (a)** Metabolic profile of MB cells before (Ctl) and after a 24 h treatment with IC_50_ of propranolol (propra) or carvedilol (carve). Mitochondrial respiration (OCR, oxygen consumption rate) and glycolytic activity (ECAR, extracellular acidification rate) were measured with the Seahorse XFe24® analyser. Values are the average of at least six independent experiments ± standard error of mean (SEM). Basal respiration **(b)**, maximal respiration **(c)**, ATP production **(d)** and glycolytic reserve **(e)** of ONS-76 cells exposed for 24 h to increasing concentrations of propranolol (propra), carvedilol (carve) or nebivolol (nebi). Values are the average of three independent experiments ± SEM, with a biological duplicate in each experiment. ECAR and OCR were normalised to cell number. *p < 0·05; **p < 0·005; ***p < 0·001.

Our previous work in triple-negative breast cancer has highlighted the ability of propranolol to affect energy metabolism pathways in tumour cells.^36^ To determine whether β-blockers disrupt the energy metabolism in MB cells, we characterised their bioenergetic profiles by measuring the mitochondrial respiration *via* the oxygen consumption rate (OCR) and the glycolytic activity *via* the extracellular acidification rate (ECAR). β-blocker treatment induced a significant drop in mitochondrial respiratory functions in ONS-76, HD-MB03, UW228-2 and DAOY cells, regardless of their initial bioenergetic status (Figure 6a and Figure S6a). Indeed, our data showed a decrease in both basal and maximal respiration after 24 h treatment with propranolol, carvedilol and nebivolol, for concentrations ranging from IC_20_ to IC_80_ (Figures 6b-c). As a result, ATP production was strongly reduced in all four MB cell lines exposed to the three β-blockers, even at the lowest concentrations (Figure 6d and Figure S6b, d, f). In addition, we demonstrated that incubation with propranolol, carvedilol and nebivolol led to a decrease in the glycolytic reserve in ONS-76, HD-MB03, DAOY and UW228-2 cells (Figure 6e and Figure S6c, e, g). Thus, treatment with β-blockers results in a metabolic catastrophe that deprives MB cells of their adaptive bioenergetics capacities.

**Figure 7:**
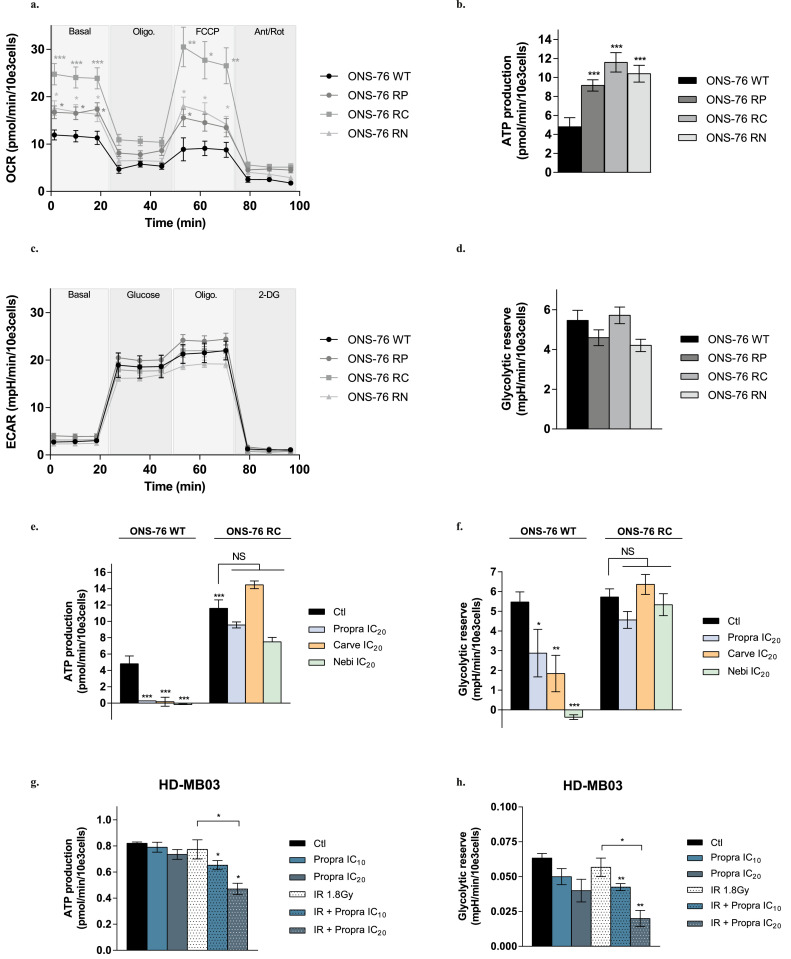
**MB cells resistant to β-blockers are not sensitive to the treatment-mediated alteration of energy metabolism.** Mitochondrial oxygen consumption rate (OCR) **(a)** and ATP production **(b)** in β-blocker sensitive cells (ONS-76 WT) and in three β-blocker resistant cells (ONS-76 RP, ONS-76 RC, ONS-76 RN), measured using the Seahorse XFe24® analyser. Glycolytic extracellular acidification rate (ECAR) **(c)** and glycolytic reserve **(d)** in β-blocker sensitive cells (ONS-76 WT) and in β-blocker resistant cells (ONS-76 RP, ONS-76 RC, ONS-76 RN), measured using the Seahorse XFe24® analyser. ATP production **(e)** and glycolytic reserve **(f)** measured with the Seahorse XFe24® analyser in sensitive and carvedilol resistant cells (ONS-76 RC) exposed to IC_20_ of propranolol (propra), carvedilol (carve) or nebivolol (nebi) for 24 h. ATP production **(g)** and glycolytic reserve **(h)** measured by using the Seahorse XFe24® analyser in HD-MB03 cells exposed for six hours to radiotherapy (IR) 1·8 Gy or IC_10_-IC_20_ of propranolol (propra) alone and their combination. All the values are the average of four independent experiments ± standard error of mean (SEM), with a biological triplicate in each experiment. Data were normalised to cell number. *p < 0·05; **p < 0·005; ***p < 0·001.

To better understand the importance of bioenergetics in response to treatment, we generated β-blocker-resistant ONS-76 cells by exposing them to increasing concentrations of propranolol, carvedilol or nebivolol for 16 weeks (Figure S7a). The resulting cell lines *i.e.*, ONS-76 RP, ONS-76 RC and ONS-76 RN, were cross-resistant to all β-blockers (Figure S7b). By qRT-PCR, we showed that the expression of β-AR genes was not altered in these resistant cells (Figure S7c). RNA sequencing further indicated that cell resistance could not be explained by the downregulation of key genes of the β-AR downstream signaling and its transcriptional targets (Figure S7d). Although four of the ten isoforms of adenylate cyclase are overexpressed in ONS-76 RP cells (ADCY1, 2, 5 and 8; *p <* 0·001), this pattern of overexpression was not found in ONS-76 RC and ONS-76 RN and therefore may not be the common factor behind the cross-resistance of the cell lines to beta-blockers. Analysis of the metabolic energetic activities in the three β-blocker resistant cell lines showed that they had higher mitochondrial OCR and ATP production than the sensitive parental ONS-76 WT cells (*p <* 0·001, Figures 7a-b and Figure S7e), but no significant changes in glycolytic capacity (Figures 7c-d). However, as illustrated with ONS-76 RC cells exposed to IC_20_ of propranolol, carvedilol or nebivolol, resistant cells were able to counteract both the β-blocker-mediated suppression of ATP production by mitochondria and glycolytic reserve (Figures 7e-f).

Lastly, we measured the effects of the combination of β-blockers and IR on the bioenergetics of MB cells. This analysis was performed at short time point (6 h) and with low doses of propranolol (IC_10_ and IC_20_) to better examine the effects of the combinatorial treatment. Propranolol alone and IR alone (1·8 Gy) did not significantly affect ATP production nor glycolytic reserve of HD-MB03 cells in these conditions (Figures 7g-h). Nevertheless, IR combined with IC_20_ propranolol inhibited the two processes by 39 ± 5 % and 63 ± 11 % respectively, as compared with IR monotherapy (*p <* 0·05, Figures 7g-h). This potentiation was also confirmed in ONS-76 cells (Figure S7f-g). Our results thus support the importance of bioenergetic disturbances in the response of MB cells to β-blockers and their combination with radiotherapy.

### β-blockers enhance IR-induced oxidative stress and consequently increase DNA damage in MB cells

**Figure 8:**
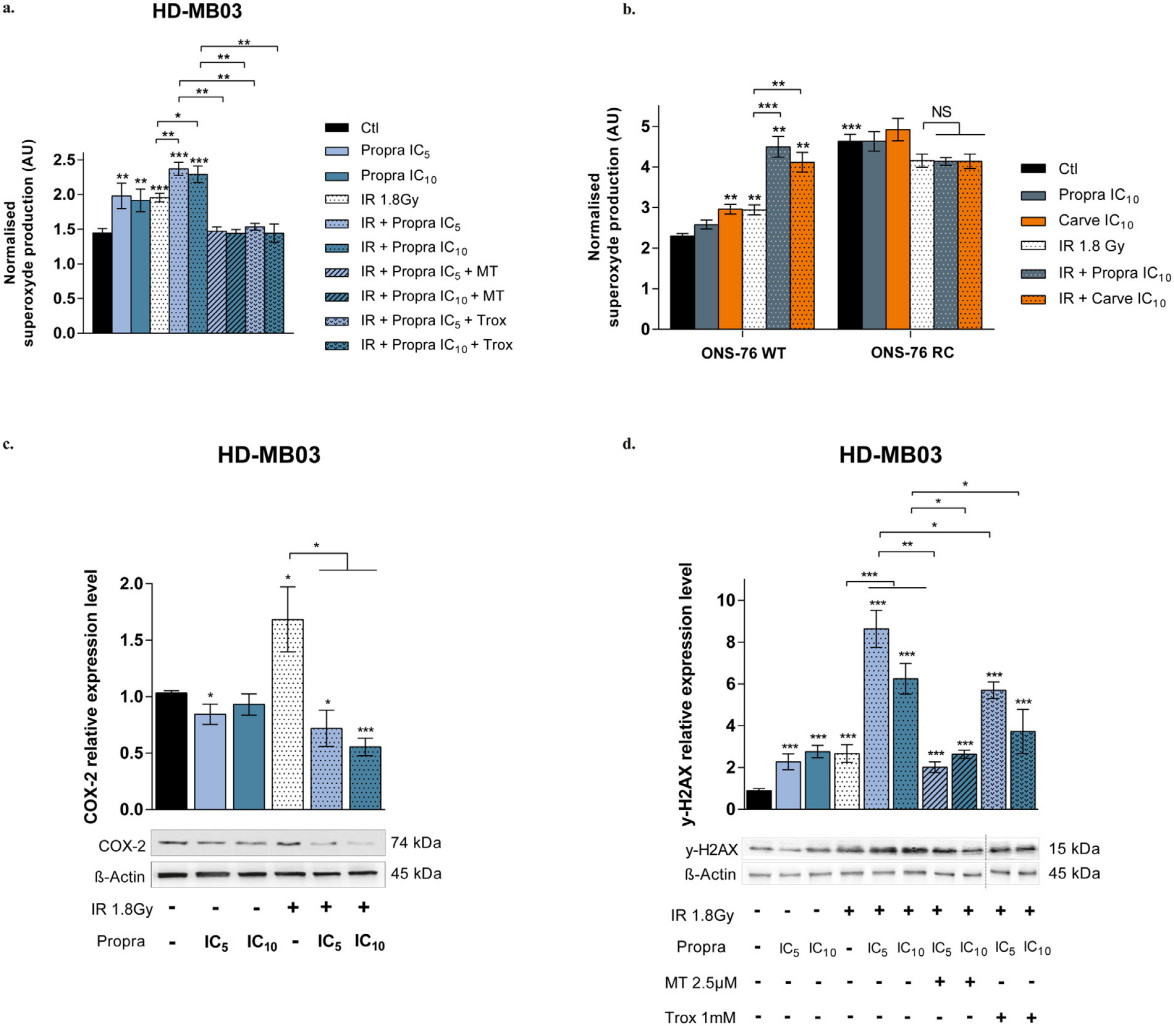
**Low concentrations of β-blockers enhanced IR-mediated MB cell oxidative stress and DNA damage. (a)** Superoxide ions production measured with WST-1 in HD-MB03 cells, six hours after treatment with radiotherapy (IR) 1·8 Gy or IC_5_-IC_10_ of propranolol (propra) alone and their combination, and with anti-oxydants (mito-TEMPO (MT) 2·5 µM or Troxerutin (Trox) 1 mM). Data were normalised to cell number. Values are the average of five independent experiments ± standard error of mean (SEM), with a biological quadruplicate in each experiment. **(b)** Superoxide ions production measured with WST-1 in β-blocker sensitive (ONS-76 WT) and carvedilol resistant ONS-76 cell lines (ONS-76 RC), six hours after treatment with radiotherapy (IR) 1·8 Gy or IC_20_ of propranolol (propra) or carvedilol (carve) alone and their combination. Data were normalised to cell number. Values are the average of four independent experiments ± SEM, with a biological quadruplicate in each experiment. Protein expression level of COX-2 **(c)** and γ-H2AX **(d)** in HD-MB03 cells exposed to radiotherapy (IR) 1·8 Gy or IC_5_-IC_10_ of propranolol (propra) alone and their combination, and with anti-oxydants (mito-TEMPO (MT) 2·5 µM or Troxerutin (Trox) 1 mM). Western blots were quantified using ImageJ™ software; data were normalised to β-Actin. Values are the average of at least five independent experiments ± SEM. *p < 0·05; **p < 0·005; ***p < 0·001.

Reactive oxygen species (ROS) are the major effectors of IR, contributing substantially to radiation-induced DNA damage and cancer cell death.^37^ Given the effects of co-treatment on mitochondrial energy metabolism, we first determined whether the combination therapy could disrupt redox balance, by assessing the superoxide ion levels. Six hours post-irradiation, an expected increase in superoxide relative level of 34 ± 4 % was observed in IR HD-MB03 cells compared to control cells (*p <* 0·001, Figure 8a). Propranolol, at 10 µM (IC_5_) and 25 µM (IC_10_), also increased the production of ROS by 36 ± 12 % and 31 ± 9 %, respectively (*p <* 0·01, Figure 8a). The combination of IR with these low doses of propranolol led to an additional upregulation of ROS levels, up to 64 ± 4 % and 59 ± 5 %, respectively (Figure 8a, *p <* 0·001). These results were confirmed with carvedilol at 5 (IC_10_) and 7·5 µM (IC_20_) in HD-MB03 cells (Figure S8a), as well as in MD 3D spheroids (Figure S8b, c). A significant overproduction of superoxide ions of 96 ± 6 % (*p <* 0·001) and 78 ± 7 % (*p <* 0·01) was also found in ONS-76 cells exposed to IR and combined with IC_10_ of propranolol or IC_10_ of carvedilol (Figure 8b). However, in the β-blocker-resistant cells ONS-76 RC, both propranolol and carvedilol were unable to amplify the effects of IR on superoxide production (Figure 8b). Potentiation of IR efficacy by the two β-blockers was also significantly reduced in these cells (Figure S8b), supporting a tight link between ROS production and response of MB cells to the combinatorial treatment.

As the overproduction of ROS may contribute to an increase in cyclooxygenase 2 (COX-2) expression, which has been associated with the acquisition of a secondary radioresistance by tumour cells,^38^ we ensured that such a feedback loop was not triggered in MB cells. By analysing COX-2 relative expression level in HD-MB03 cells 24 h after treatment, we showed that it was reduced from 1·7 ± 0·3 in 1·8 Gy irradiated cells to 0·8 ± 0·1 and 0·7 ± 0·1 in cells subjected to IR combined with IC_5_ and IC_10_ propranolol respectively (*p <* 0·05, Figure 8c). The inhibition of IR-mediated increase in COX-2 expression by the combinatorial treatment was confirmed in ONS cells (Figure S8c).

Lastly, we evaluated the phosphorylation level of H2AX, being an early response to DNA double-strand breaks that here may be caused following ROS exposure. In HD-MB03 cells, 4 h after treatment, IR triggered the expected accumulation of yH2AX, as did low concentrations of propranolol (Figure 8d). Our results also showed a significant increase in γH2AX relative level from 2·7 ± 0·4 in irradiated cells to 8·6 ± 0·9 and 6·3 ± 0·7 in cells exposed to the co-treatment with IC_5_ and IC_10_ propranolol, respectively (*p <* 0·001, Figure 8d). By scavenging the superoxide ions (Figure 8a), Mito-TEMPO (MT) counteracted the increase in γH2AX level by the combinatorial treatments – which dropped to 2·0 ± 0·4 and 2·6 ± 0·2, respectively – (*p <* 0·01 and 0·05 *vs* co-treatments, respectively, Figure 8d). Likewise, scavenging of free radicals by Troxerutin (TROX; Figure 8a) led to a significant reduction in IR-mediated γH2AX accumulation (*p <* 0·05 *vs* co-treatments, Figure 8d). Taken together, our results suggest that β-blockers can specifically modulate mitochondrial bioenergetics and ROS production in MB cells, thus priming them for IR-induced oxidative stress and DNA damage. Our results therefore show that the efficacy of the combination of IR with β-blockers is, at least in part, based on a strong inhibition of MB cell bioenergetics, linked to the triggering of an endogenous oxidative stress.

## Discussion

In recent years, many advances have been made in the management of children with MB. Nevertheless, a real concern remains the long-term sequelae due to the early exposure to toxic treatments.^7^^,^^10^ Drug repurposing appears to be a major tool to rapidly find effective and well-tolerated therapeutic approaches in oncology.^11^^,^^13^ It might especially be an alternative strategy to manage rare cancers such as paediatric tumours. Cardiovascular regulators, anti-helminthic drugs and non-steroidal anti-inflammatory drugs have recently shown to reduce MB tumour cell progression *in vitro* and *in vivo*.^39^ Here, we evaluated in MB models propranolol, carvedilol and nebivolol, which are lipophilic β-blockers that can cross the blood-brain barrier, and enter the cerebrospinal fluid and intracranial tissue.^40^

Our results showed that the three β-blockers potentiate the efficacy of IR in a panel of MB cells, PDX cells and spheroid micromasses, including those poorly responsive to radiation. These results are consistent with the recent study from Chaudhary *et al*., that described a propranolol-mediated sensitisation to IR in non-small cell lung cancer cells *in vitro*.^20^ Enhanced effectiveness of IR at reducing the growth of gastric adenocarcinoma *in vivo* when combined with propranolol was also shown recently.^23^ Retrospective clinical studies have also shown that the combination of ß-blockers and radiotherapy did not result in increased toxicities in patients with lung cancer^20^^,^^41^^,^^42^ and brain tumors such as meningioma.^24^ The interest of combining radiotherapy with β-blockers is further supported by the fact that ß-blockers are largely known to be good brain protectors that can be used for instance after head trauma including in children.^43^^,^^44^

In response to the ever-increasing need to find alternatives to animal experimentation, we have developed an innovative organotypic cerebellum model in which MB tumour progression has been analysed over time. These *ex vivo* tissue cultures are described as highly relevant models to study the evolution of pathologies and to test their response to different therapeutic strategies, including in MB.^45^ We further showed that the dose of IR can be significantly reduced while maintaining treatment efficacy in MB cells by adding β-blockers. As the severity of cognitive damages in patients correlates with radiation doses,^10^^,^^46^ this suggests that combining β-blockers with IR may help limit treatment side effects.

One of the advantages of repurposing β-blockers as anti-cancer agents is that they can be translated to the clinic without the need for extensive preclinical studies, including *in vivo* experiments. For instance, propranolol was first used in a clinical setting in combination with metronomic chemotherapy in patients before it was later confirmed to be active *in vivo* in mouse models.^17^^,^^47^^,^^48^ In addition, an ongoing clinical trial (NCT04682158) exploring the combination of propranolol with chemo-radiation is based on *in vitro* experiments and retrospective clinico-epidemiological experience in patients who received β-blockers for non-cancer purposes in combination.^49^ Another potential example is based on multiple myeloma for which clinical trials have been completed (NCT02420223) or recently initiated (NCT02420223) without myeloma-specific *in vivo* data but based but again on *in vitro* and clinic-epidemiological experiments.^50^ The results of the present article can thus provide a strong basis for initiating an early phase clinical trial.

The literature is divided regarding the mechanisms responsible for the anti-tumour properties of β-blockers. Inhibition of the β-adrenergic signaling pathway has been suggested to be involved in propranolol activity in pancreatic cancer cells.^51^ Studies in angiosarcoma cells provide a good illustration of the conflicting hypotheses. Amaya *et al.* proposed the involvement of the β-adrenergic pathway in the mechanism of action of propranolol,^52^^,^^53^ whereas a recent study by Overman *et al.* argued the opposite and showed a key role for the SOX18 protein in the response to the β-blocker.^52^^,^^53^ In the paediatric tumours neuroblastoma and hemangioma, the results agree that β-ARs are not responsible for the anti-tumour efficacy of β-blockers, showing that the R-enantiomer of propranolol – which has very low affinity for β-ARs – has the same efficacy as the S-enantiomer that is highly affine for the receptors.^53^, ^54^, ^55^ Although patient MB biopsies showed detectable β-AR expression, we demonstrated herein that their silencing did not alter the efficacy of propranolol, carvedilol and nebivolol in MB cells, suggesting that the efficacy of β-blockers in MB cells may not result from the inhibition of the canonical targets.

We and others have reported that propranolol-exposed cancer cells were sensitised to the metabolic stress induced by metformin, rapamycin, 2-deoxy-D-glucose or dichloroacetate.^36^^,^^56^, ^57^, ^58^ Here, we showed that the activity of the β-blockers in MB cells was driven by a rapid disruption of the mitochondrial bioenergetics, which led to a sustained accumulation of ROS. This is consistent with the alteration of the mitochondrial fusion/fission balance that we previously observed in neuroblastoma cells treated with propranolol.^18^ The significance of cancer cell energy metabolism in response to β-blockers is further strengthened by the fact that the lack of impact on mitochondrial and glycolytic pathways results in resistance of MB cells to these repurposed drugs.

Efficacy of radiation therapy relies on its ability to cause DNA breaks and to subsequently trigger cell death. The DNA damages mainly result from the generation of ROS, such as superoxide and hydroxyl radicals, during H_2_O radiolysis.^37^ In the present study, we showed that β-blockers potentiate IR-mediated DNA damages in MB cells by increasing superoxide accumulation. Our results are consistent with the fact that pharmacologic depletion of glutathione, which belongs to the cell antioxidant system, significantly results in radiosensitisation of cancer stem cells.^59^ Recently, Gd-doped titania nanoparticules that target mitochondria to enhance ROS accumulation were also shown to sensitise breast cancer cells to radiotherapy-induced apoptosis *in vitro* and *in vivo*.^60^ Increasing ROS levels in MB tumour cells during radiotherapy may thus significantly enhance the efficiency and decrease the dosage of radiation.

COX-2 overexpression has been associated with resistance to IR in prostate, lung and oral squamous cancer cells.^61^, ^62^, ^63^ Conversely, COX-2 inhibitors can synergise with IR in inducing apoptosis,^63^^,^^64^ including in MB stem-like cells.^65^^,^^66^ COX-2 inhibition has been suggested as a potential strategy in MB to decrease the production of prostaglandin E2 (PGE2) and ultimately promote tumour cell death.^67^ Here, we showed that propranolol prevented the increase in COX-2 expression mediated by IR, but the involvement of the PGE2 pathway in improving response of MB cells to combinatorial therapy remains to be better characterised.

To conclude, our work highlights the interest of channeling the ability of β-blockers to inhibit mitochondrial bioenergetics to design new therapeutic combinations with radiotherapy that lower the dose while maintaining anti-tumour activity. Given the few druggable molecular targets identified in non-WNT MB and the fact that young age of patients limits treatment options, our work proposes an alternative approach in which drug repurposing could be quickly translated to the clinic to improve the efficacy of radiotherapy and/or decrease its toxicity.

## Contributors

Conceptualisation: E.P., M.C. and N.A. Formal analysis: M.R., J.T., M.M., P.P., M.-P.M., E.A.-C., M., R.A. and D.W. Funding acquisition: N.A., M.R. and M.C. Methodology: J.T., C.K., D.B., L.P., P.P., M.-P.M., E.A.-C., R.A. and V.B. Project administration: M.C., S.C., O.A. and N.A. Supervision: M.C. and N.A. Validation: M.R., O.A.,J.T., M.L.G., P.P., E.P., D.B., M.-P.M. and M.M. Writing, review & editing: M.R., M.C., E.P., M.L.G., N.A., D.W. and J.T. All authors have read and approved the final manuscript.

## Data sharing statement

Our RNA-seq information and raw data from ONS-76 cells are publicly available in GEO database (GEO accession numbers: GSE191165). The other data and materials are available from the corresponding authors upon reasonable request.

## Declaration of interests

The authors have declared no conflict of interest.

## Appendix Supplementary materials

Image, application 1

Image, application 2

Image, application 3

Image, application 4
