# Supplementary material for: Beta-blockers disrupt mitochondrial bioenergetics and increase radiotherapy efficacy independently of beta-adrenergic receptors in medulloblastoma
Source: eBioMedicine. 2022 Jul 8;82:104149. doi: 10.1016/j.ebiom.2022.104149 (PMC9283511; doi:10.1016/j.ebiom.2022.104149)

Supplementary Figure 1

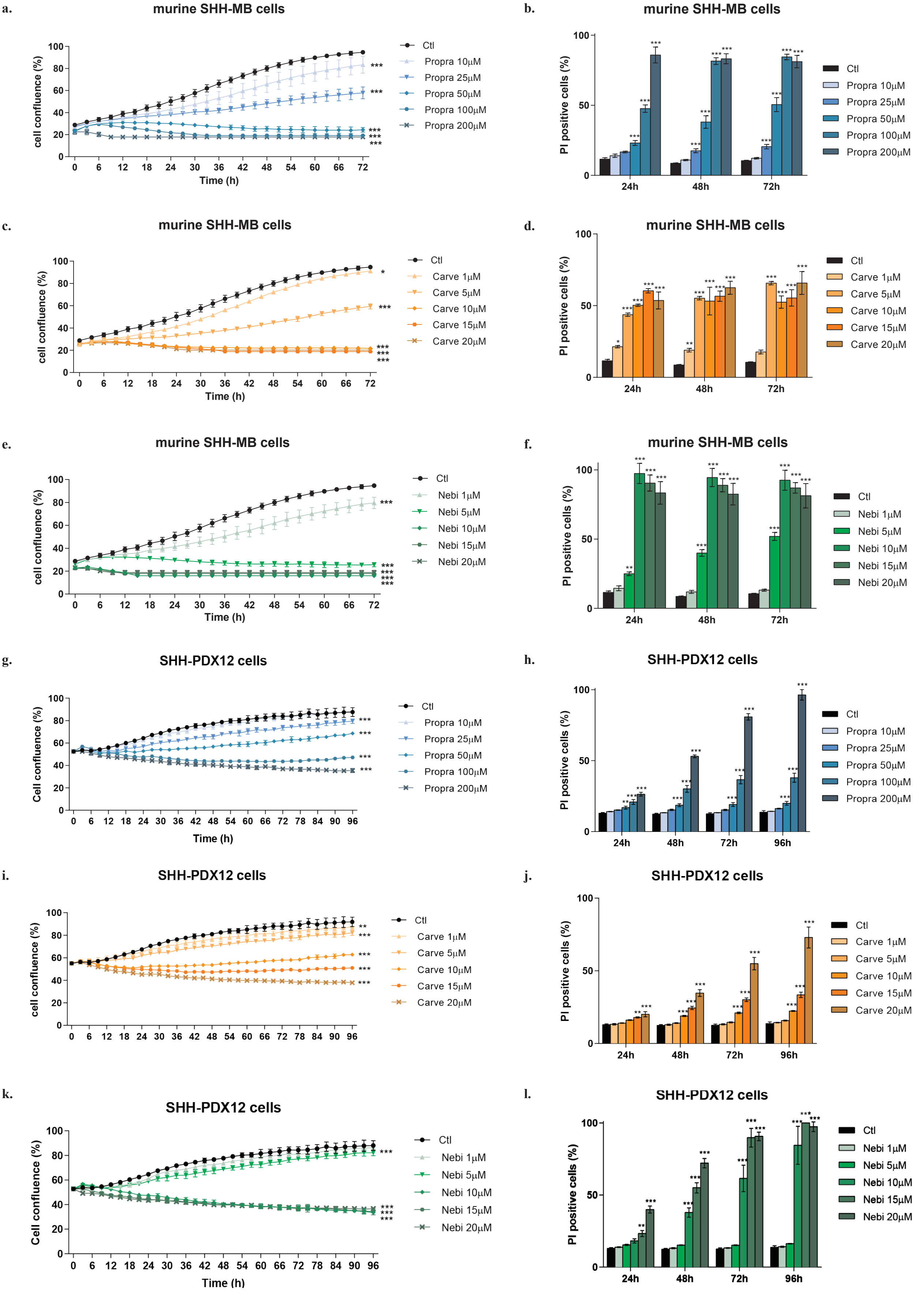

Supplementary Figure 2

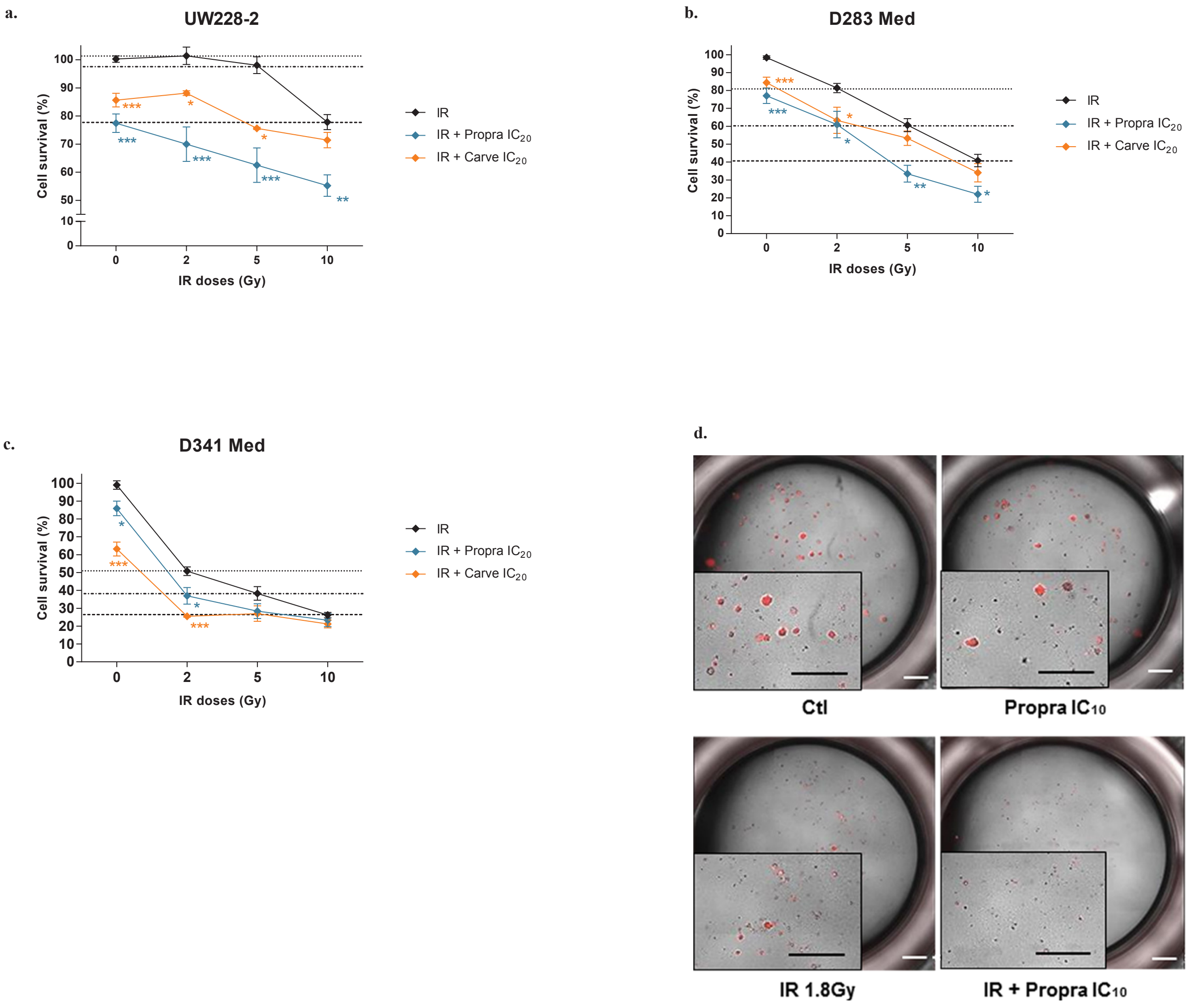

Supplementary Figure 3

a.

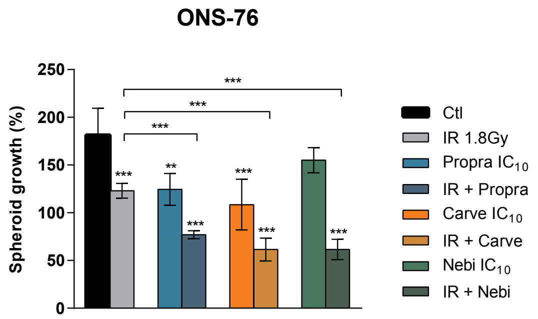

b.

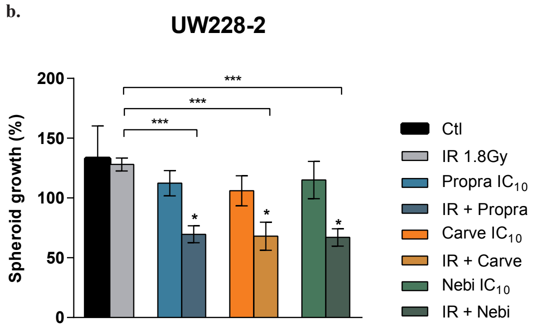

c.

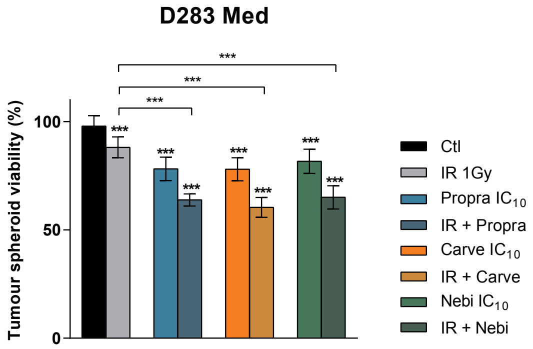

d.

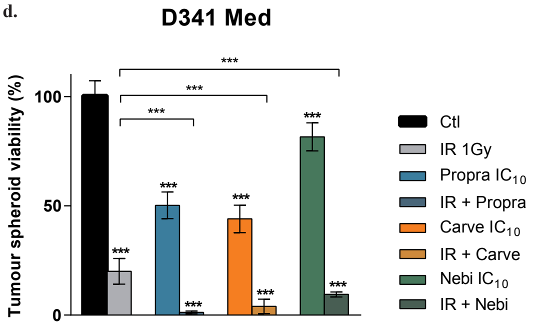

e.

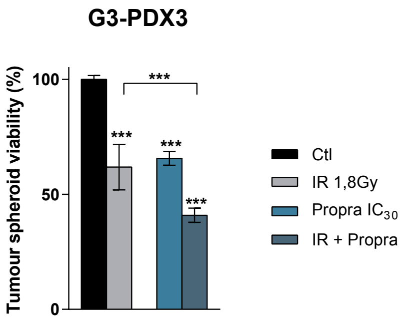

Supplementary Figure 4

a.

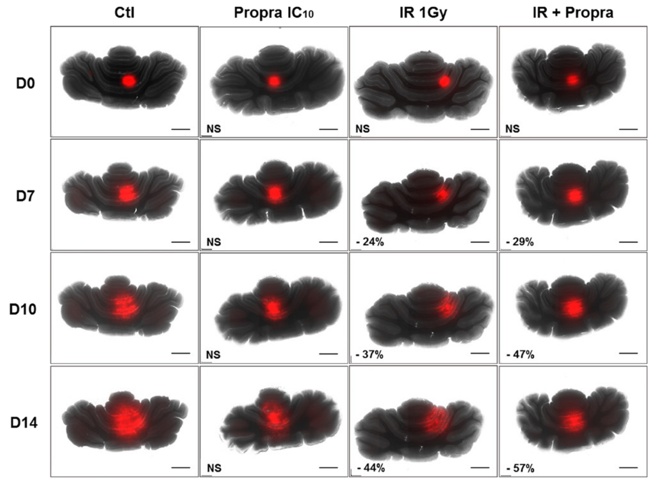

b.

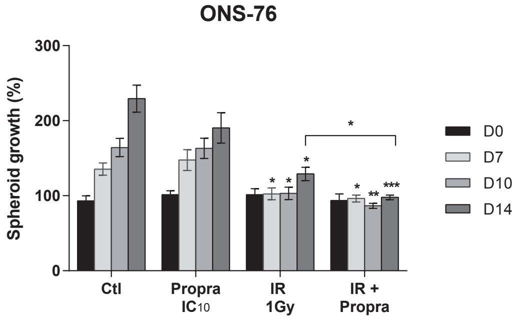

c.

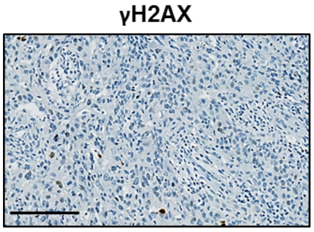

Supplementary Figure 5

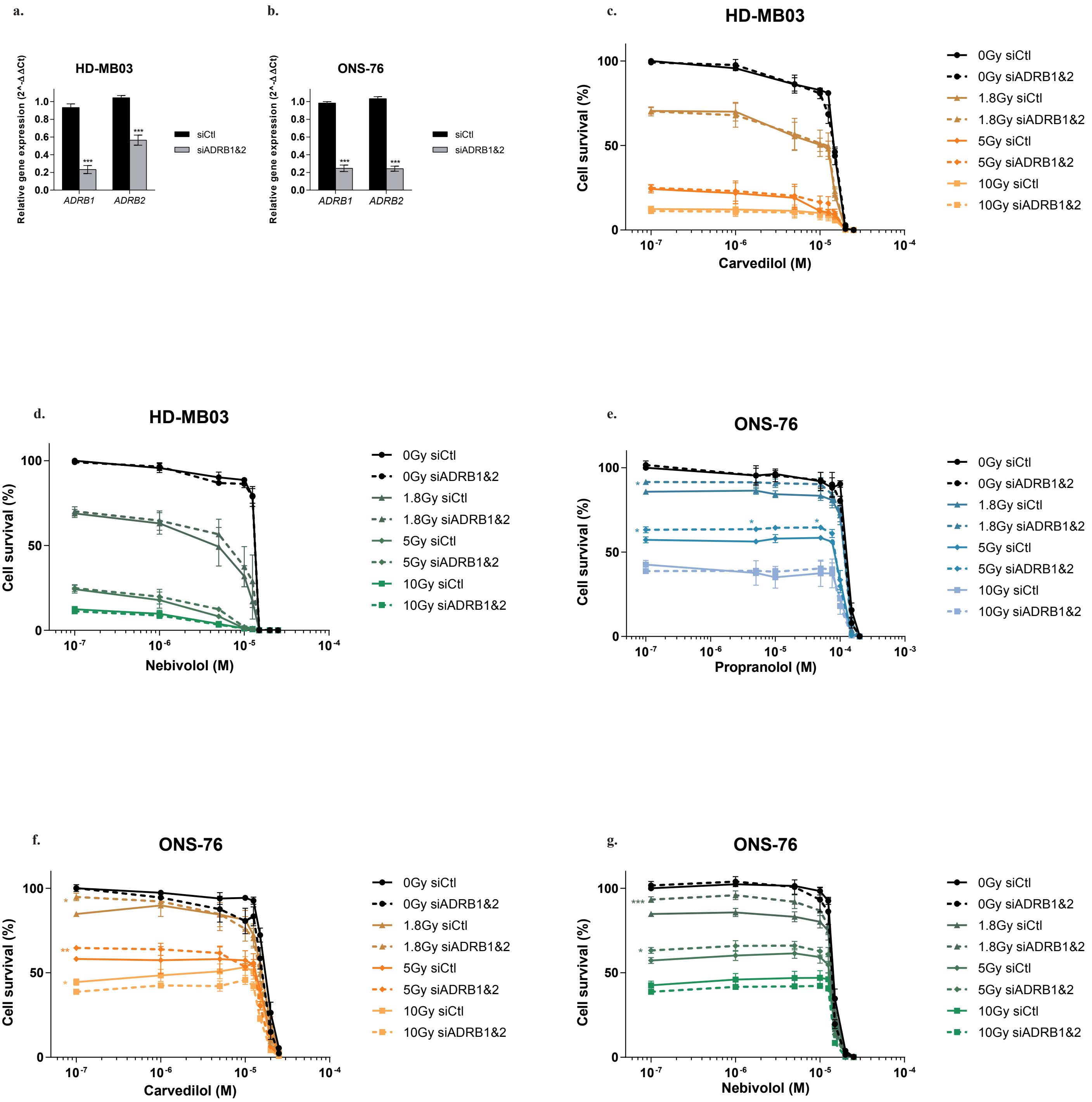

Supplementary Figure 6

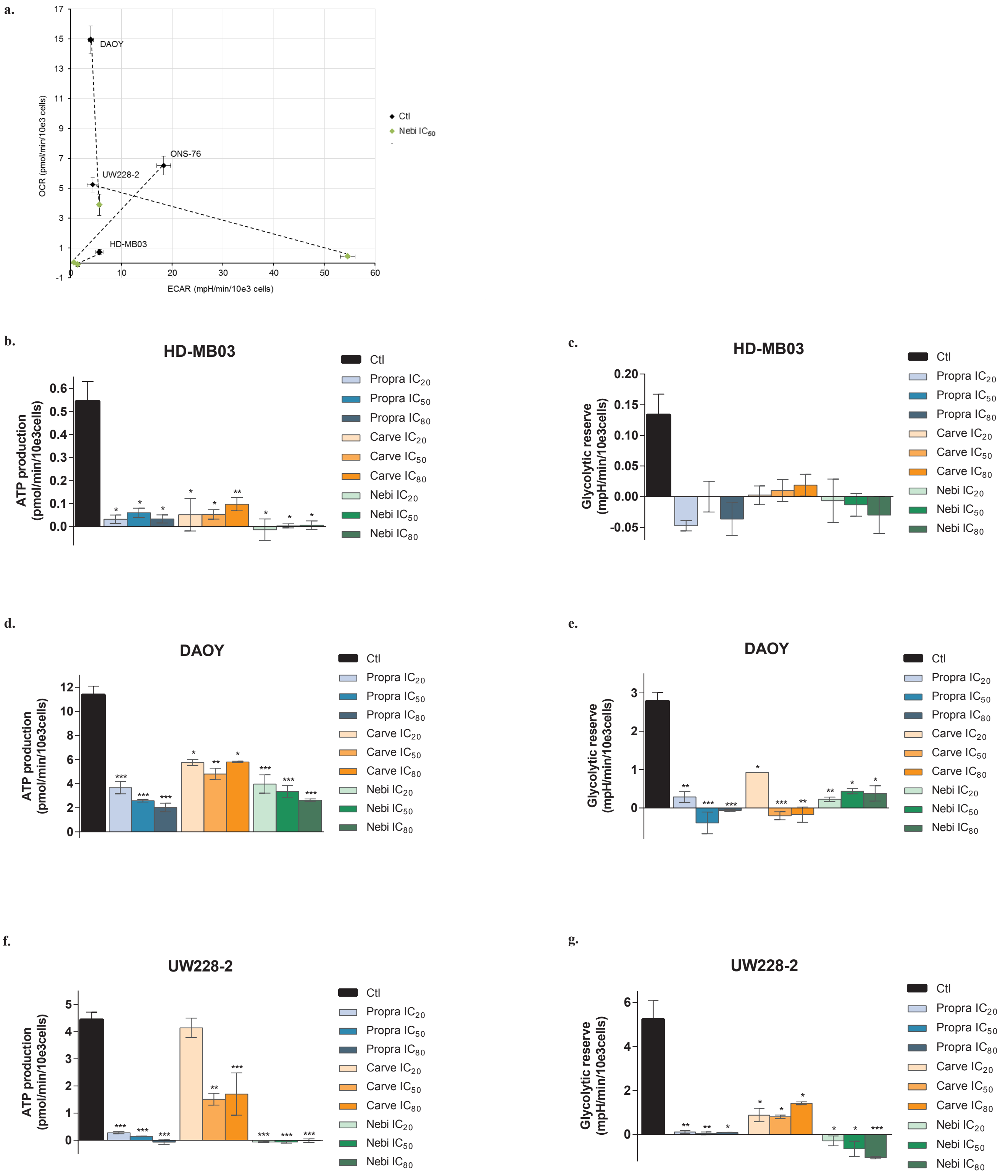

Supplementary Figure 7

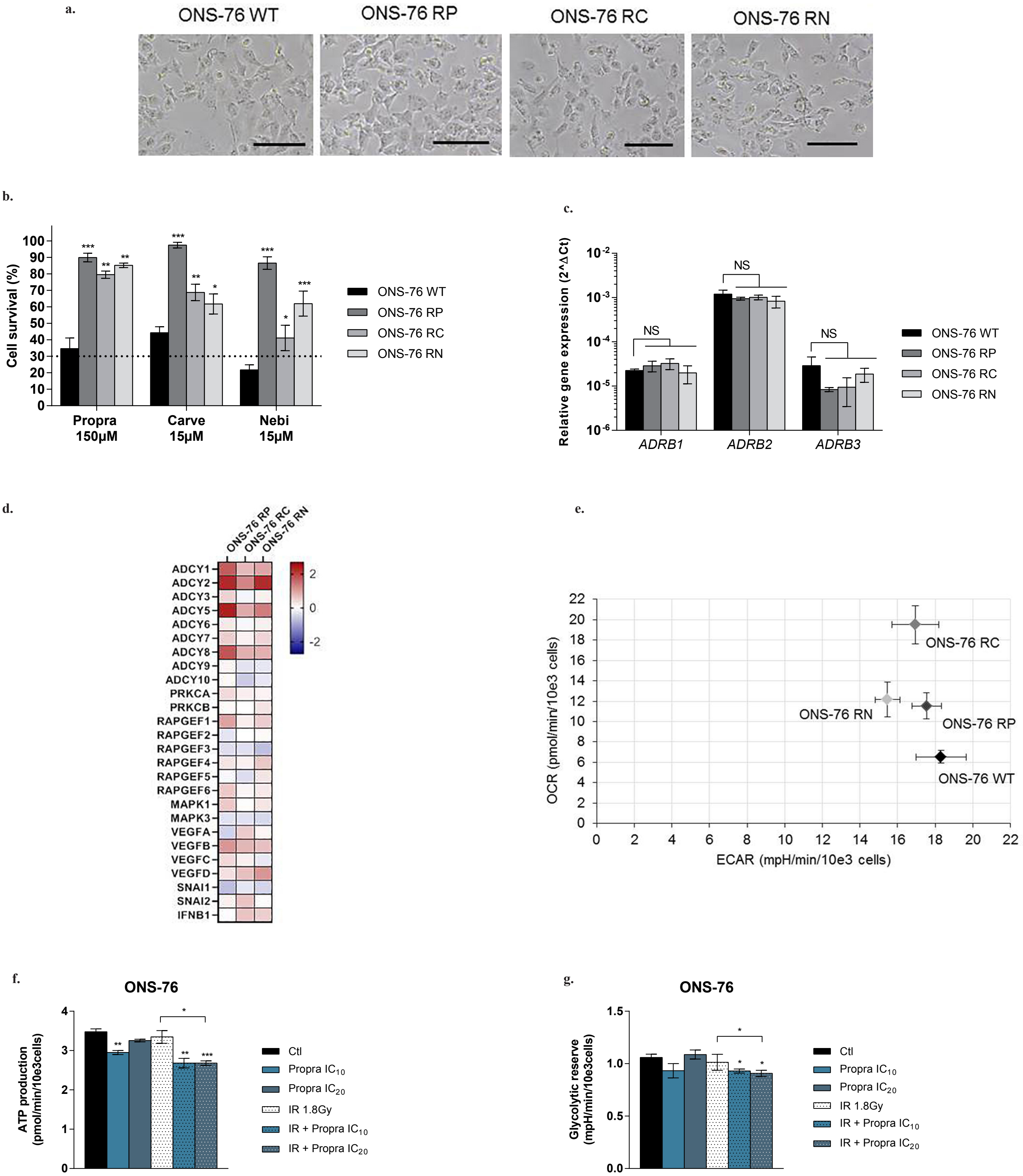

Supplementary Figure 8

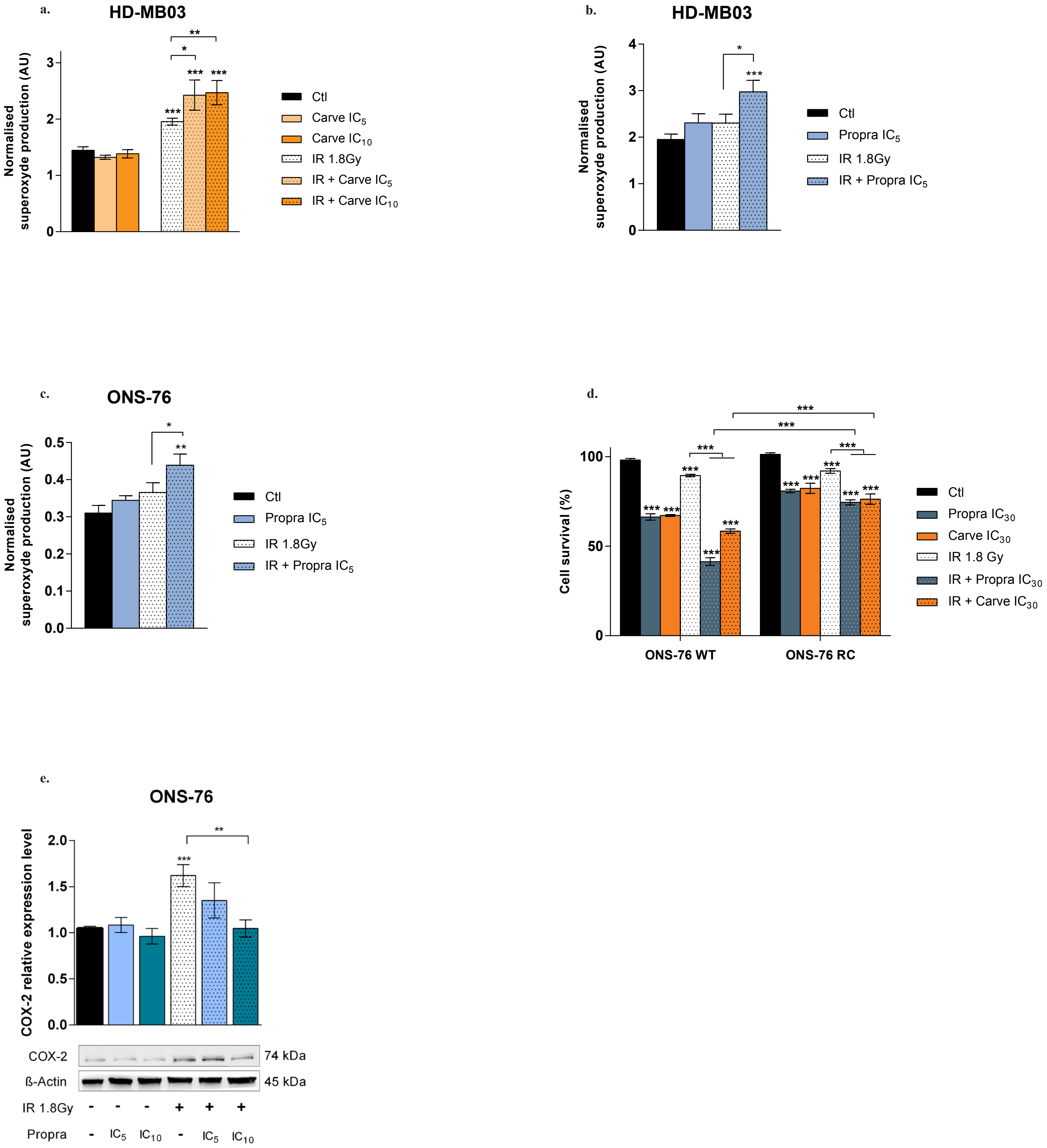

Supplement: Supplementary file 1 [file mmc1.pdf]
