# Supplementary material for: Beta-blockers disrupt mitochondrial bioenergetics and increase radiotherapy efficacy independently of beta-adrenergic receptors in medulloblastoma
Source: eBioMedicine. 2022 Jul 8;82:104149. doi: 10.1016/j.ebiom.2022.104149 (PMC9283511; doi:10.1016/j.ebiom.2022.104149)
Supplement: Supplementary file 2 [file mmc2.pdf]

Supplementary Figures - Captions

Table 1: IC_{50} values of propranolol, carvedilol and nebivolol after 72 h of treatment of non-Wnt human MB cells (DAOY, UW228-2, HD-MB03, ONS-76, D283 Med, D341 Med), mice MB cells (murine SHH-MB) and PDX-isolated cells (G3-PDX7 and SHH-PDX12), determined by GraphPad Prism software. Values are the average of at least three independent experiments ± SD.

Table 2: yH2AX positive cells (% +/- SD) in the cerebellum of the organotypic models. Quantification was made by microscopic analysis in the Neuropathology Department. *P* values > 0·05 indicate no significant variations.

Supplementary Figure 1: Inhibition of MB cell survival by β-blockers. Cell confluence assessment over time by using the IncuCyte® live cell analysis system, in murine SHH-MB cells after treatment with increase concentrations of propranolol (propra) **(a)**, carvedilol (carve) **(c)** and nebivolol (nebi) **(e)**. Quantification of cell death at 24 h, 48 h and 72 h of treatment with increase concentrations of propranolol **(b)**, carvedilol **(d)** and nebivolol **(f)** by staining in murine SHH-MB cells with propidium iodure (PI). Cell confluence assessment over time by using the IncuCyte® live cell analysis system, in SHH-PDX12 cells after treatment with increase concentrations of propranolol (propra) **(g)**, carvedilol (carve) **(i)** and nebivolol (nebi) **(j)**. Quantification of cell death at 24 h, 48 h, 72 h and 96 h of treatment with increase concentrations of propranolol **(h)**, carvedilol **(j)** and nebivolol **(l)** by staining SHH-PDX12 cells with propidium iodure (PI). All values are the average of at least three independent experiments ± SD. $^{*}p < 0{\cdot}05$; $^{**}p < 0{\cdot}005$; $^{***}p < 0{\cdot}001$.

Supplementary Figure 2: Increase of IR efficacy by β-blockers in MB cells. UW228-2 **(a)**, D283 Med **(b)** and D341 Med **(c)** cell survival measured by the Alamar Blue assay after treatment with IC_{20} of propranolol (propra) or carvedilol (carve) alone and combined to radiotherapy (IR) 2, 5 and 10 Gy. Values are the average of at least three independent experiments ± SEM. **(d)** Representative images of HD-MB03 cell colony formation, acquired with the JuLi™ Stage system, 10 days after initiation of treatment with a IC_{10} of propranolol or radiotherapy (IR) 1·8 Gy alone and their combination. Scale bars: 500 µm. $^{*}p < 0{\cdot}05$; $^{**}p < 0{\cdot}005$; $^{***}p < 0{\cdot}001$.

Supplementary Figure 3: β-blockers enhance IR activity in 3D spheroids of MB cell lines and MB PDX cells. For five consecutive days, the 3D tumour micromasses were exposed to daily low doses (IC_{10}) of propranolol, carvedilol and nebivolol alone or combined to radiotherapy (IR) 1·8 Gy. ONS-76 **(a)** and UW228-2 **(b)** spheroid growth was measured by acquisition of the DsRed signal with the PHERAstar microplate reader after 10 days of treatment with IC_{10} of propranolol (propra), carvedilol (carve) and nebivolol (nebi) alone or combined with radiotherapy (IR) 1·8 Gy. Values are the average of at least four independent experiments ± SD. D283 Med **(c)** and D341 Med **(d)** spheroid viability was assessed by using Alamar Blue after 7 days of treatment with IC_{10} of propranolol (propra), carvedilol (carve) and nebivolol (nebi) alone or combined with radiotherapy (IR) 1·8 Gy. Values are the average of at least six independent experiments ± SD. **(e)** G3-PDX3 spheroid viability was measured by using the CellTiter-Glo® assay after 72 h of treatment with IC_{30} of propranolol (propra) alone or combined with radiotherapy (IR) 1·8 Gy. Values are the average of at least three independent experiments ± SD. $^{*}p < 0{\cdot}05$; $^{**}p < 0{\cdot}005$; $^{***}p < 0{\cdot}001$.

Supplementary Figure 4: Benefits of daily low concentrations of β-blockers combined to IR in *ex-vivo* MB organotypic model. For five consecutive days, the organotypic cerebellar co-cultures were exposed to daily low doses (IC_{10}) of propranolol alone or combined to radiotherapy (IR) 1·8 Gy. **(a)** Representative pictures, acquired with the JuLi™ Stage live imaging system, of DsRed-expressing ONS-76 tumour micromasses grafted in slices of healthy cerebellum. Scale bars: 1 mm. Results were expressed as percentage of growth inhibition in treated *vs* control organotypic models (Ctl). **(b)** ONS-76 tumour growth was measured over 14 days by acquisition of the DsRed signal with the PHERAstar microplate reader (well-scanning mode) (n=4). Values are the average of independent experiments ± SEM. **(c)** Positive control of γH2AX immunostaining in breast carcinoma tissue. Scale bars: 100 µm. $^{*}p < 0{\cdot}05$; $^{**}p < 0{\cdot}005$; $^{***}p < 0{\cdot}001$.

Supplementary Figure 5: β-Adrenergic receptors (β-AR) are not involved in the response of MB cells to β-blockers. Relative gene expression level of *ADRB1* and *ADRB2* in HD-MB03 **(a)** and ONS-76 **(b)** cells after transfection of siRNA control or siRNA *ADRB1&2*. *ADRB1* and *ADRB2* mRNA was quantified by qRT-PCR using *GAPDH* as housekeeping gene (n=4); calculation has been done by the 2^-ΔΔCt method. Values are the average of independent experiments ± SEM. Cell viability analysis by using the Alamar Blue assay after 72 h of treatment with radiotherapy (IR) 1·8, 5 and 10 Gy alone and combined with increase concentrations of carvedilol **(c)** or nebivolol **(d)** in HD-MB03 cells transfected with siRNA control (siCtl) or siRNA *ADRB1&2*. Same experiments with propranolol **(e)**, carvedilol **(f)** or nebivolol **(g)** in ONS-76 cells transfected with siRNA control

(siCtl) or siRNA *ADRB1&2*. Values are the average of at least three independent experiments ± SEM. *$p < 0{\cdot}05$; **$p < 0{\cdot}005$; ***$p < 0{\cdot}001$.

Supplementary Figure 6: β-blockers inhibit MB cell energy metabolism. (a) Metabolic profile of MB cells before (Ctl) and after a 24 h treatment with IC_{50} of nebivolol (nebi). Mitochondrial respiration (OCR, oxygen consumption rate) and glycolytic activity (ECAR, extracellular acidification rate) were measured with the Seahorse XFe24® analyser. Values are the average of at least six independent experiments ± SEM. ATP production determined using the Seahorse XFe24® analyser in HD-MB03 **(b)**, DAOY **(d)** and UW228-2 **(f)** cells exposed for 24 h to increasing concentrations of propranolol (propra), carvedilol (carve) or nebivolol (nebi). Glycolytic reserve determined using the Seahorse XFe24® analyser in HD-MB03 **(c)**, DAOY **(e)** and UW228-2 **(g)** cells exposed for 24 h to increasing concentrations of propranolol (propra), carvedilol (carve) or nebivolol (nebi). Data were normalised to cell number. Values are the average of at least three independent experiments ± SEM. *$p < 0{\cdot}05$; **$p < 0{\cdot}005$; ***$p < 0{\cdot}001$.

Supplementary Figure 7: MB cells resistant to β-blockers have an unchanged β-AR signaling pathway but exhibit greatly enhanced mitochondrial energy metabolism. (a) Representative pictures of β-blocker sensitive (ONS-76 WT) and resistant (ONS-76 RP, ONS-76 RC, ONS-76 RN) cells, acquired with the Nikon Eclipse TS100 fluorescence microscope. Scale bars: 100 µm. **(b)** Survival of β-blocker sensitive and resistant cells quantified by using the Alamar Blue assay after 72 h of treatment with propranolol (propra) 150 µM, carvedilol (carve) 15 µM and nebivolol (nebi) 15 µM. Values are the average of at least three independent experiments ± SEM. **(c)** Relative gene expression of *ADRB1*, *ADRB2* and *ADRB3* in the ONS-76 β-blocker sensitive and resistant cells, quantified by qRT-PCR using *GAPDH* as housekeeping gene (n=3). Calculation has been done by the 2^-ΔCt method. Values are the average of independent experiments ± SEM. **(d)** Heatmap of log2 Fold Change for genes coding for β-adrenergic (β-AR) pathway genes. Differential gene expression was analysed between the three β-blocker resistant cell lines compared to β-blocker sensitive cells (n=3). Values are the average of independent experiments. **(e)** Metabolic profile of the four β-blocker sensitive and resistant ONS-76 cell lines. Mitochondrial respiration (OCR, oxygen consumption rate) and glycolytic activity (ECAR, extracellular acidification rate) were measured with the Seahorse XFe24® analyser. Data were normalised to cell number. Values are the average of at least ten independent experiments ± SEM. ATP production **(f)** and glycolytic reserve **(g)** measured by using the Seahorse XFe24® analyser in ONS-76 cells exposed for six hours to radiotherapy (IR) 1·8 Gy or IC_{10}-IC_{20} of propranolol (propra) alone and their combination. Data were normalised to cell number. Values are the average of at least three independent experiments ± SEM. *$p < 0{\cdot}05$; **$p < 0{\cdot}005$; ***$p < 0{\cdot}001$.

Supplementary Figure 8: Low concentrations of β-blockers increase IR-mediated MB cell oxidative stress and DNA damage. (a) Superoxide ions production measured with WST-1 in HD-MB03 cells, six hours after treatment with radiotherapy (IR) 1·8 Gy or IC_{10}-IC_{20} of carvedilol (carve) alone and their combination. Data were normalised to cell number. Values are the average of at least four independent experiments ± SEM. Superoxide ions production measured with WST-1 in HD-MB03 **(b)** and ONS-76 **(c)** spheroids, six hours after treatment with radiotherapy (IR) 1·8 Gy or IC_5 of propranolol (propra) alone and their combination. Data were normalised to cell number. Values are the average of at least four independent experiments ± SEM. **(d)** Cell survival analysis in β-blocker sensitive ONS-76 (ON-76 WT) and in carvedilol resistant ONS-76 (ONS-76 RC) cell lines by Alamar Blue assay after 72 h of treatment with radiotherapy (IR) 1·8 Gy or IC_{30} of propranolol (propra) or carvedilol (carve) alone and their combination. Values are the average of at least four independent experiments ± SEM. **(e)** Protein expression level of COX-2 in ONS-76 cells exposed to radiotherapy (IR) 1·8 Gy or IC_5-IC_{10} of propranolol (propra) and their combination. Western blots were quantified using ImageJ™ software. Data were normalised to β-Actin. Values are the average of at least three independent experiments ± SEM. *$p < 0{\cdot}05$; **$p < 0{\cdot}005$; ***$p < 0{\cdot}001$.
