# Supplementary figures and images for: Beta-blockers disrupt mitochondrial bioenergetics and increase radiotherapy efficacy independently of beta-adrenergic receptors in medulloblastoma
Source: eBioMedicine. 2022 Jul 8;82:104149. doi: 10.1016/j.ebiom.2022.104149 (PMC9283511; doi:10.1016/j.ebiom.2022.104149)

Western blots from Fig 8C

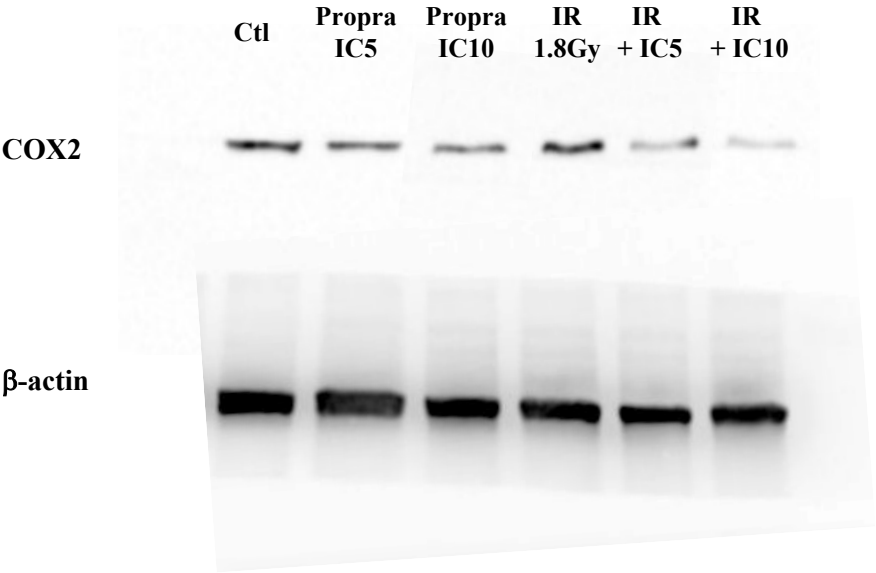

Western blots from Fig 8D

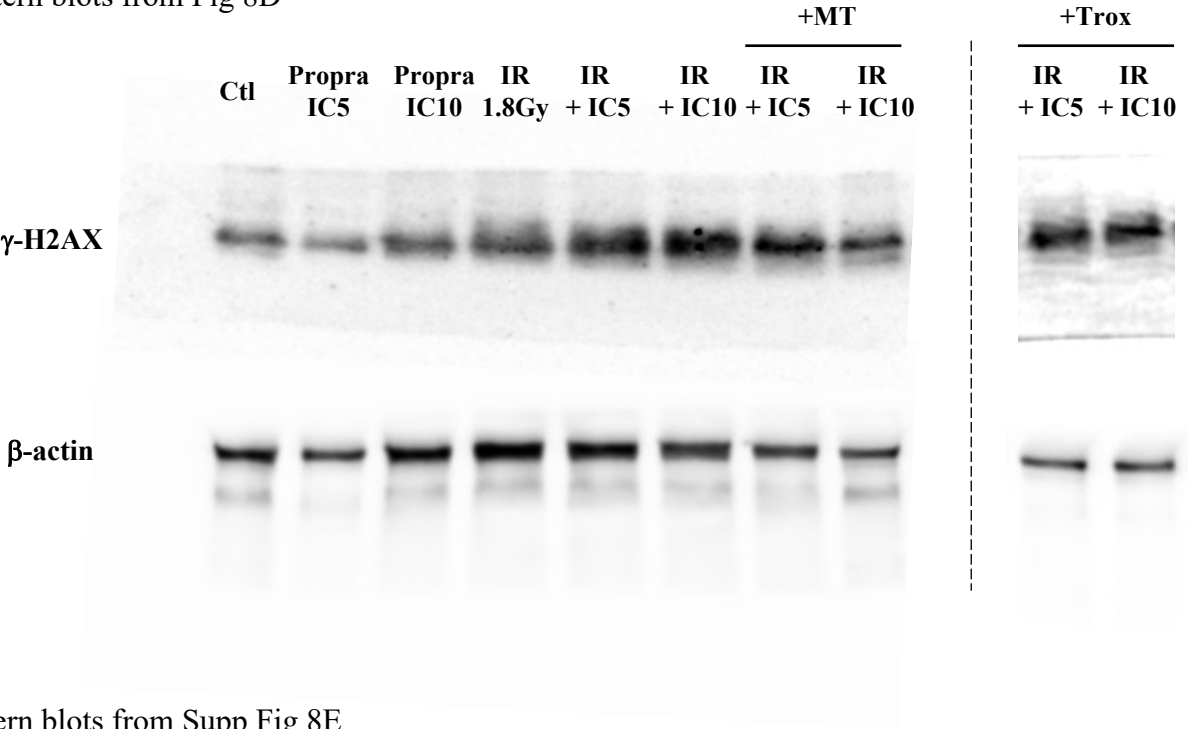

Western blots from Supp Fig 8E

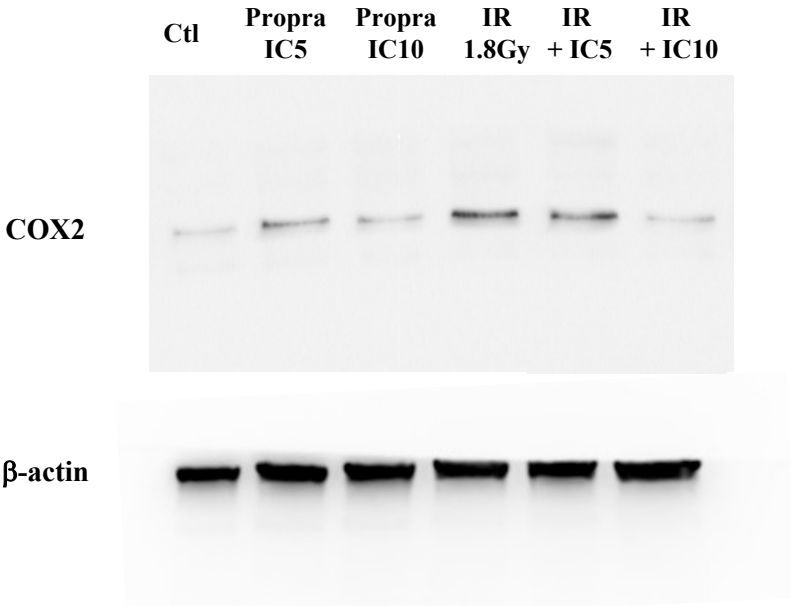

Supplement: Supplementary file 3 [file mmc3.pdf]
