# Supplementary material for: Beta-blockers disrupt mitochondrial bioenergetics and increase radiotherapy efficacy independently of beta-adrenergic receptors in medulloblastoma
Source: eBioMedicine. 2022 Jul 8;82:104149. doi: 10.1016/j.ebiom.2022.104149 (PMC9283511; doi:10.1016/j.ebiom.2022.104149)
Supplement: Supplementary file 4 [file mmc4.pdf]

Example of a mycoplasma test on medulloblastoma cells and *ex vivo* cerebellum in culture.

	HD-MB03	D341	ONS-76	Cerebellum	Positive control
**Level of ATP in the sample before the addition of the MycoAlert® substrate (Read A)**	1223	1239	559	350	627
**Level of ATP in the sample after the addition of the MycoAlert® substrate (Read B)**	340	403	180	236	25276
**Ratio Read B / Read A**	0,278	0,325	0,320	0,670	40,310

Validation of the siRNAs directed against β1- or β2- adrenergic receptors over time.

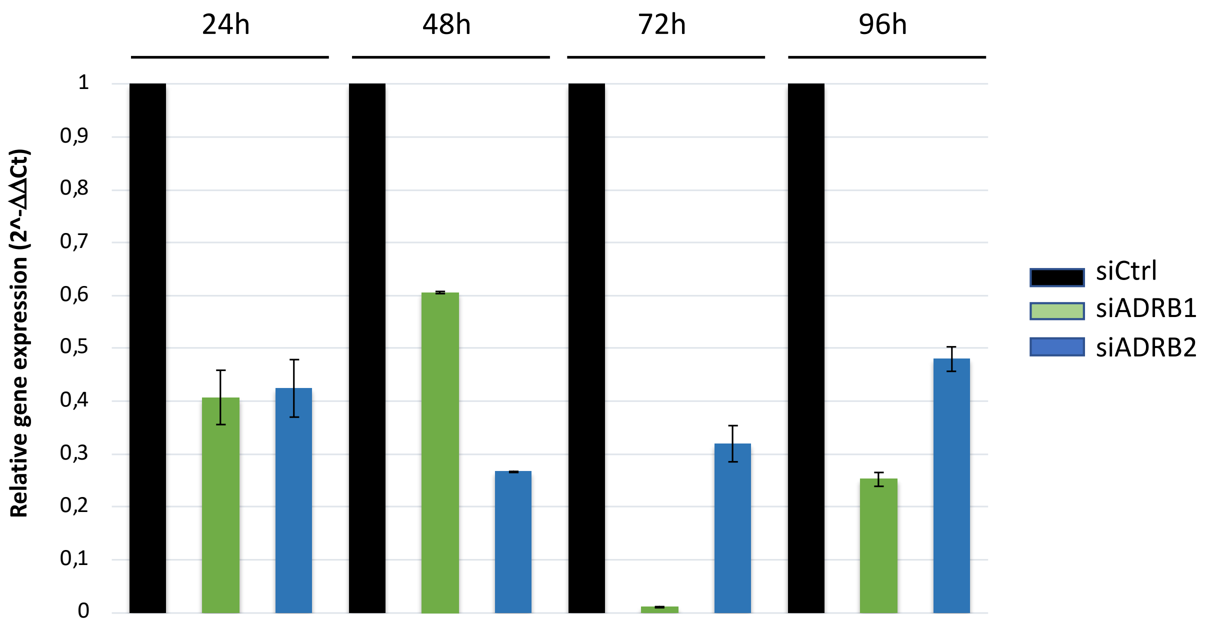

CELL PANEL

BRAIN CANCER CELL PANEL

The Brain Cancer Cell Panel (ATCC® TCP-1017™) comprises 4 brain cancer cell lines with varying degrees of genetic complexity. Each culture contains genomic mutations in one or more of the following genes according to the Sanger COSMIC database: TP53, CDKN2A, PTEN, and NF1. The table below provides more information for the cell lines included in this panel.

ATCC® No.	Name	Tumor Source	Tumorigenic	Histology	Mutant Gene	Zygosity	Gene Sequence	Protein Sequence
CRL-2060™	PFSK-1	primary	yes	primitive neuroectodermal tumor (PNET)	TP53	homozygous	c.823T>G	p.C275G
CRL-1620™	A172	primary	no	glioma	CDKN2A	homozygous	c.1_471del471	p.0?
					PTEN	homozygous	c.165_1212del1048	p.R55fs*1
HTB-12™	SW1088	primary	yes	glioma	CDKN2A	homozygous	c.1_471del471	p.0?
					PTEN	homozygous	c.165_1212del1048	p.R55fs*1
					TP53	homozygous	c.817C>T	p.R273C
HTB-186™	Daoy	primary	yes	medulloblastoma	CDKN2A	homozygous	c.1_471del471	p.0?
					NF1	homozygous	c.61_4835del4775	p.?
					TP53	homozygous	c.725G>T	p.C242F

The mutation data was obtained from the Sanger Institute Catalogue Of Somatic Mutations In Cancer web site, http://www.sanger.ac.uk/cosmic Bamford *et al* (2004) The COSMIC (Catalogue of Somatic Mutations in Cancer) database and website. Br J Cancer, 91,355-358. ATCC and The Sanger Institute provide these data in good faith, but make no warranty, express or implied, nor assumes any legal liability or responsibility for any purpose for which the data are used. The ATCC trademark and trade name, any and all ATCC catalog numbers, and any other trademarks listed are trademarks of the American Type Culture Collection unless indicated otherwise. ATCC products are intended for laboratory research only. They are not intended for use in humans, animals or diagnostics.

©2022 American Type Culture Collection. The ATCC trademark and trade name, and any other trademarks listed in this publication are trademarks owned by the American Type Culture Collection unless indicated otherwise.

These products are for laboratory use only. Not for human or diagnostic use. ATCC products may not be resold, modified for resale, used to provide commercial services or to manufacture commercial products without prior ATCC written approval.

CERTIFICATE OF ANALYSIS

ATCC® Number: **HTB-185™**

Lot Number: **62114305**

Name: **D283 Med**

Description: **Medulloblastoma**

Species: **Human (*Homo sapiens*)**

Volume/Ampoule: **Approximately 1 mL**

Date Frozen: **12/2/13**

Recovery: **A T-25 setup at a seeding density of 4.0 x 10^5 viable cells/mL is ready to subculture in 2 days.**

A T-75 setup at a seeding density of 4.0 x 10^5 viable cells/mL is ready to subculture in 5 days.

Product Format: **Cells cryopreserved in the appropriate cryopreservation medium**

Expiration Date: **Not applicable**

Storage Conditions: **Vapor phase of liquid nitrogen**

Test / Method	Specification	Result
**Ampule passage number**	Report results	Unknown
**Population doubling level (PDL)**	Report results	N/A
**Total cells/ampoule**<br>(Cell count using Trypan Blue stain method)	Report results	8.7 x 10^6 total cells/ampoule
**Post-freeze viability**<br>(Cell count using Trypan Blue stain method)	≥ 50.0%	74.8%
**Growth properties**<br>(Visual observation method)	Mixed – adherent and/or suspension	Mixed- adherent and/or suspension
**Morphology**<br>(Visual observation method)	Epithelial-like and/or rounded*	Epithelial-like and/or rounded
**Test for mycoplasma contamination**<br>Hoechst DNA stain (indirect) method<br>Agar culture (direct) method	 None detected<br>None detected	 None detected<br>None detected
**Species determination: COI assay (interspecies)**	Human	Human

ATCC (American Type Culture Collection)
P.O. Box 1549
Manassas, VA 20108 USA
www.atcc.org

800-638-6597 or 703-365-2700
Fax: 703-365-2750
E-mail: tech@atcc.org
or contact your local distributor

CONFIDENTIAL AND PROPRIETARY
This document contains proprietary information which may not be reproduced, transcribed, or conveyed in any way or for any purpose without the prior written consent of ATCC.
Template Doc ID: 31194 Template Revision: 3 Template Effective Date: 01/31/2013

CERTIFICATE OF ANALYSIS

ATCC® Number: **HTB-185™**

Lot Number: **62114305**

**Species determination: STR analysis (intraspecies)**	**Human (Unique DNA Profile)**<br>TH01: 7<br>D5S818: 11<br>D13S317: 8, 10<br>D7S820: 10<br>D16S539: 11<br>CSF1PO: 9, 12<br>Amelogenin: X, Y<br>vWA: 16, 18<br>TPOX: 8, 11	**Human (Unique DNA Profile)**<br>TH01: 7<br>D5S818: 11<br>D13S317: 8, 10<br>D7S820: 10<br>D16S539: 11<br>CSF1PO: 9, 12<br>Amelogenin: X, Y<br>vWA: 16, 18<br>TPOX: 8, 11
**Sterility test (BacT/ALERT 3D)**<br>iAST bottle (aerobic) at 32°C<br>iNST bottle (anaerobic) at 32°C	 No growth<br>No growth	 No growth<br>No growth
**Human pathogenic virus testing**<br>(PCR-based assay for HIV, HepB, HPV, EBV, and CMV)	Report results	HIV – none detected<br>HepB – none detected<br>HPV – none detected<br>EBV – none detected<br>CMV – none detected

* Epithelial-like: Any adherent cells of a polygonal shape with clear, sharp boundaries between them.

Jo Salisbury

Digitally signed by Jo Salisbury
DN: cn=Jo Salisbury, o=ATCC, ou=Quality Assurance, email=jsalisbury@atcc.org, c=US
Date: 2014.02.04 10:11:28 -05'00'

Quality Assurance Specialist; Quality Assurance

ATCC hereby represents and warrants that the material provided under this certificate is pure and has been subjected to the tests and procedures specified and that the results described, along with any other data provided in this certificate, are true and correct to the best of the company's knowledge and belief. This certificate does not extend to the growth and/or passage of any living organism or cell line beyond what is supplied within the container received from ATCC.

This product is intended to be used for laboratory research use only. It is not intended for use in humans, animals, or for diagnostics. Appropriate Biosafety Level (BSL) practices should always be used with this material. Refer to the Product Information Sheet for instructions on the correct use of this product.

ATCC products may not be resold, modified for resale, used to provide commercial services, or to manufacture commercial products without prior written agreement from ATCC.

The ATCC trademark and trade name and any and all ATCC catalog numbers are trademarks of the American Type Culture Collection.

© 2010 ATCC. All rights reserved.

ATCC (American Type Culture Collection)
P.O. Box 1549
Manassas, VA 20108 USA
www.atcc.org

800-638-6597 or 703-365-2700
Fax: 703-365-2750
E-mail: tech@atcc.org
or contact your local distributor

CONFIDENTIAL AND PROPRIETARY
This document contains proprietary information which may not be reproduced, transcribed, or conveyed in any way or for any purpose without the prior written consent of ATCC.
Template Doc ID: 31194 Template Revision: 3 Template Effective Date: 01/31/2013

CERTIFICATE OF ANALYSIS

**ATCC® Number:**	**HTB-187™**
**Lot Number:**	**61259186**
**Name:**	**D341 Med**
**Description:**	**Medulloblastoma**
**Species:**	**Human (*Homo sapiens*)**
**Volume/Ampule:**	**Approximately 1 mL**
**Date Frozen:**	**12/17/2012**
**Recovery:**	**A T-25 setup at a dilution of 1:10 is ready to subculture in 7 days.**<br>**A T-75 setup at a dilution of 1:15 is ready to subculture in 12 days.**
**Product Format:**	**Cells cryopreserved in the appropriate cryopreservation medium**
**Expiration Date:**	**Not applicable**
**Storage Conditions:**	**Vapor phase of liquid nitrogen**

Test / Method	Specification	Result
**Ampule passage number**	Report results	Unknown
**Population doubling level (PDL)**	Report results	Not applicable
**Total cells/ampule**<br>(Cell count using Trypan Blue stain method)	Report results	5.7×10^6 total viable cells
**Post-freeze viability**<br>(Cell count using Trypan Blue stain method)	Report results	66.6%
**Growth properties**<br>(Visual observation method)	Suspension	Suspension
**Morphology**<br>(Visual observation method)	Rounded	Rounded
**Test for mycoplasma contamination**<br>Hoechst DNA stain (indirect) method<br>Agar culture (direct) method	 None detected<br>None detected	 None detected<br>None detected
**Species determination: COI assay (interspecies)**	Human	Human

ATCC (American Type Culture Collection)
P.O. Box 1549
Manassas, VA 20108 USA
www.atcc.org

800-638-6597 or 703-365-2700
Fax: 703-365-2750
E-mail: tech@atcc.org
or contact your local distributor

CONFIDENTIAL AND PROPRIETARY
This document contains proprietary information which may not be reproduced, transcribed, or conveyed in any way or for any purpose without the prior written consent of ATCC.
Template Doc ID: 31194 Template Revision: 3 Template Effective Date: 01/31/2013

CERTIFICATE OF ANALYSIS

ATCC® Number: **HTB-187™**

Lot Number: **61259186**

**Species determination: STR analysis (intraspecies)**	**Human (Unique DNA Profile)**<br>D5S818: 11,12<br>D13S317: 11,13<br>D7S820: 9,13<br>D16S539: 12,14<br>vWA: 17,18<br>TH01: 6,9.3<br>Amelogenin: X,Y<br>TPOX: 8,11<br>CSF1PO: 10,11	**Human (Unique DNA Profile)**<br>D5S818: 11,12<br>D13S317: 11,13<br>D7S820: 9,13<br>D16S539: 12,14<br>vWA: 17,18<br>TH01: 6,9.3<br>Amelogenin: X,Y<br>TPOX: 8,11<br>CSF1PO: 10,11
**Sterility test (BacT/ALERT 3D)**<br>iAST bottle (aerobic) at 32°C<br>iNST bottle (anaerobic) at 32°C	No growth<br>No growth	No growth<br>No growth
**Human pathogenic virus testing**<br>(PCR-based assay for HIV, HepB, HPV, EBV, and CMV)	Report results	HIV – None detected<br>HepB – None detected<br>HPV – None detected<br>EBV – None detected<br>CMV – None detected

Chelsea Clegg

Digitally signed by Chelsea Clegg
DN: cn=Chelsea Clegg, o=ATCC, ou=Quality Assurance, email=cclegg@atcc.org, c=US
Date: 2013.02.21 10:53:38 -05'00'

Quality Assurance Specialist

ATCC hereby represents and warrants that the material provided under this certificate is pure and has been subjected to the tests and procedures specified and that the results described, along with any other data provided in this certificate, are true and correct to the best of the company's knowledge and belief. This certificate does not extend to the growth and/or passage of any living organism or cell line beyond what is supplied within the container received from ATCC.

This product is intended to be used for laboratory research use only. It is not intended for use in humans, animals, or for diagnostics. Appropriate Biosafety Level (BSL) practices should always be used with this material. Refer to the Product Information Sheet for instructions on the correct use of this product.

ATCC products may not be resold, modified for resale, used to provide commercial services, or to manufacture commercial products without prior written agreement from ATCC.

The ATCC trademark and trade name and any and all ATCC catalog numbers are trademarks of the American Type Culture Collection.

© 2010 ATCC. All rights reserved.

ATCC (American Type Culture Collection)
P.O. Box 1549
Manassas, VA 20108 USA
www.atcc.org

800-638-6597 or 703-365-2700
Fax: 703-365-2750
E-mail: tech@atcc.org
or contact your local distributor

CONFIDENTIAL AND PROPRIETARY
This document contains proprietary information which may not be reproduced, transcribed, or conveyed in any way or for any purpose without the prior written consent of ATCC.
Template Doc ID: 31194 Template Revision: 3 Template Effective Date: 01/31/2013
